# The centriolar satellite protein Cfap53 facilitates formation of the zygotic microtubule organizing center in the zebrafish embryo

**DOI:** 10.1242/dev.198762

**Published:** 2022-08-18

**Authors:** Sven Willekers, Federico Tessadori, Babet van der Vaart, Heiko H. Henning, Riccardo Stucchi, Maarten Altelaar, Bernard A. J. Roelen, Anna Akhmanova, Jeroen Bakkers

**Affiliations:** 1 Hubrecht Institute-KNAW, Utrecht 3584 CT, The Netherlands; 2 Cell Biology, Neurobiology and Biophysics, Department of Biology, Faculty of Science, Utrecht University, Utrecht 3584 CH, The Netherlands; 3 Equine Sciences, Department Clinical Sciences, Faculty of Veterinary Medicine, Utrecht University, Utrecht 3584 CM, The Netherlands; 4 Biomolecular Mass Spectrometry and Proteomics, Bijvoet Center for Biomolecular Research and Utrecht Institute for Pharmaceutical Sciences, Utrecht University, Utrecht 3584 CH, The Netherlands; 5 Embryology, Anatomy and Physiology, Department Clinical Sciences, Faculty of Veterinary Medicine, Utrecht University, Utrecht 3584 CT, The Netherlands; 6 Department of Pediatric Cardiology, Division of Pediatrics, University Medical Center Utrecht, Utrecht 3584 EA, The Netherlands

**Keywords:** Ccdc11, Cfap53, Centriolar satellites, MTOC, Cell division, Maternal, Paternal, Zebrafish

## Abstract

In embryos of most animal species, the zygotic centrosome is assembled by the centriole derived from the sperm cell and pericentriolar proteins present in the oocyte. This zygotic centrosome acts as a microtubule organizing center (MTOC) to assemble the sperm aster and mitotic spindle. As MTOC formation has been studied mainly in adult cells, very little is known about the formation of the zygotic MTOC. Here, we show that zebrafish (*Danio rerio*) embryos lacking either maternal or paternal Cfap53, a centriolar satellite protein, arrest during the first cell cycle. Although Cfap53 is dispensable for sperm aster function, it aids proper formation of the mitotic spindle. During cell division, Cfap53 colocalizes with γ-tubulin and with other centrosomal and centriolar satellite proteins at the MTOC. Furthermore, we find that γ-tubulin localization at the MTOC is impaired in the absence of Cfap53. Based on these results, we propose a model in which Cfap53 deposited in the oocyte and the sperm participates in the organization of the zygotic MTOC to allow mitotic spindle formation.

## Abstract (precis)

**Summary:** Cfap53 colocalizes with centrosomal and centriolar satellite proteins at the MTOC during the first zygotic cell division where it facilitates the recruitment of γ-tubulin and formation of the mitotic spindle.

## INTRODUCTION

The animal embryo must undergo multiple rounds of cell divisions to form a multicellular organism composed of hundreds of different cell types. During each cell division, microtubule organizing centers (MTOCs) participate in the formation of the mitotic spindle required to segregate the chromosomes into the two daughter cells. In dividing animal cells, the centrosome is the main MTOC. Centrosomes are composed of a pair of centrioles surrounded by a complex protein structure consisting of microtubule nucleating and anchoring proteins called the pericentriolar material (PCM) (reviewed by Doxsey, 2001; Gould and Borisy, 1977).

In most animal oocytes, centrosomes are eliminated during oogenesis (Schatten et al., 1989; Szollosi et al., 1972). After fertilization, centrosomes are reassembled, which requires the interaction between the centrioles present in the sperm cell and maternal factors present in the oocyte (Holy and Schatten, 1997; Yabe et al., 2007). The sperm cell contains a pair of centrioles termed proximal and distal centrioles, but most centrosomal components are eliminated from the sperm cell (reviewed by Schatten, 1994; Sutovsky and Schatten, 1999). Subsequently, after fertilization, the proximal centriole recruits centrosomal components that are deposited maternally in the oocyte to form a functional zygotic centrosome (reviewed by Schatten, 1994). One of the best characterized centrosomal components that is recruited to the MTOC after fertilization is γ-tubulin. It is part of the γ-tubulin ring complex (γTuRC) that serves as a template for nucleating microtubules (reviewed by Kollman et al., 2011). How γTuRC recruitment is facilitated towards the MTOC is still unclear. *In vitro* studies have shown that both microtubule-based active transport and passive diffusion contribute to centrosomal γTuRC recruitment (Dammermann and Merdes, 2002; Félix et al., 1994; Khodjakov and Rieder, 1999; Moritz et al., 1998; Quintyne et al., 1999; Schnackenberg et al., 1998; Stearns and Kirschner, 1994; Young et al., 2000). In rodents, the sperm centriole is lost during spermatogenesis (Schatten et al., 1986; Woolley and Fawcett, 1973). However, MTOCs are still formed by recruitment of centrosomal components independently of centrioles (Gueth-Hallonet et al., 1993).

During early cleavage stages, zebrafish embryos have large MTOCs in which PCM components do not form a well-defined structure, but appear as cytoplasmic foci (Lindeman and Pelegri, 2012; Rathbun et al., 2020). This specific organization of the MTOC could support a mechanism that enables the mitotic spindle to span the large embryonic cells. The establishment of this unique MTOC after fertilization is probably highly coordinated and dependent on maternal and/or paternal factors present in the fertilized embryo.

Forward genetic screens in zebrafish are an unbiased approach to identify parental factors that have an important biological role in early development (Pelegri and Mullins, 2016). Several zebrafish and medaka mutants have been identified via this approach and proven to be very informative for the identification and characterization of some of these maternal and paternal factors (Abrams et al., 2020; Dekens et al., 2003; Inoue et al., 2017; Yabe et al., 2007). The zebrafish gene *cellular atoll* (*cea*) encodes for the centriole duplication factor Sas-6 (Yabe et al., 2007). Maternal *cea* (*sass6*) mutant embryos proceed through the first cell division but show mitotic defects from the second cell division onwards. Paternal *cea* mutant embryos have a delayed first cell division, but cell division proceeds normally thereafter. The zebrafish gene *futile cycle* encodes the lymphoid-restricted membrane protein (Lrmp); in maternal *fue* (*lrmp*) mutant embryos, the male and female pronuclei fail to fuse (Dekens et al., 2003; Lindeman and Pelegri, 2012). In normal conditions, the two pronuclei fuse to one zygote nucleus in a process called nuclear congression. Nuclear congression requires the formation of a microtubule aster that nucleates from the proximal centriole and attaches to the pronuclei to bring them together, a process that fails in *fue* mutants (Dekens et al., 2003; Lindeman and Pelegri, 2012). In mouse embryos, nuclear congression appears different as it is facilitated by two bipolar spindles formed by two clusters of MTOCs around each pronucleus (Reichmann et al., 2018). The medaka (*Oryzias latipes*) maternal WD40 repeat-containing protein, Wdr8 (the orthologue of human WRAP73), is important during MTOC assembly (Inoue et al., 2017). Maternal zygotic *wdr8* (*wrap73*) mutant embryos form multipolar mitotic spindles resulting in chromosome alignment errors. With its WD40-containing domain, Wdr8 interacts with the centriolar satellite protein SSX2IP (Inoue et al., 2017). Additional maternal effect mutations that affect cell division in the early embryo have been identified via a recent forward genetic screen, expanding the molecular-genetic understanding of parental contribution in early embryonic development further (Abrams et al., 2020).

Centriolar satellites have been identified as non-membranous granules that surround the centrosomes (Dammermann and Merdes, 2002; Kubo et al., 1999; Odabasi et al., 2019; Prosser and Pelletier, 2020). The best known marker for centriolar satellites is PCM1 as it is the first centriolar satellite protein that was identified (Balczon et al., 1994; Kubo et al., 1999; Kubo and Tsukita, 2003). Since then, many more proteins have been identified as components of centriolar satellites, including the cilia- and flagella-associated protein Cfap53 (also known as Ccdc11). Many of the identified centriolar satellites contain coiled-coil domains, including PCM1 and Cfap53 (Balczon et al., 1994; Kubo et al., 1999; Silva et al., 2016).

To gain insight into the formation of the MTOC and the role of centriolar satellite proteins herein, we have studied the zebrafish *cfap53* mutant. We and others have previously shown that the zebrafish *cfap53* gene is expressed in ciliated organs of the embryo, including the Kupffer's vesicle, which is the zebrafish left-right organizer (Bakkers et al., 2009; Hirokawa et al., 2006). Zebrafish and mouse *cfap53/Cfap53* mutant embryos develop normally but show organ laterality defects due to compromised cilia function in the left-right organ, which resembles patients with homozygous *CFAP53* mutations (Ide et al., 2020; Narasimhan et al., 2015; Noël et al., 2016; Perles et al., 2012; Silva et al., 2016). Zebrafish homozygous *cfap53* mutants are adult viable and, in this study, we find that embryos lacking either maternal or paternal Cfap53 arrest in the first cell division. In the absence of Cfap53, the formation of the mitotic spindle is affected. We report that Cfap53 interacts with centriolar satellite proteins and centrosomal components, and plays a role in the localization of γ-tubulin to the zygotic MTOC. These findings reveal a novel role for maternal and paternal Cfap53 during zygotic MTOC formation in the zebrafish embryo.

## RESULTS

### Cfap53 has a maternal and paternal role in embryonic cell division

**Fig. 1.**
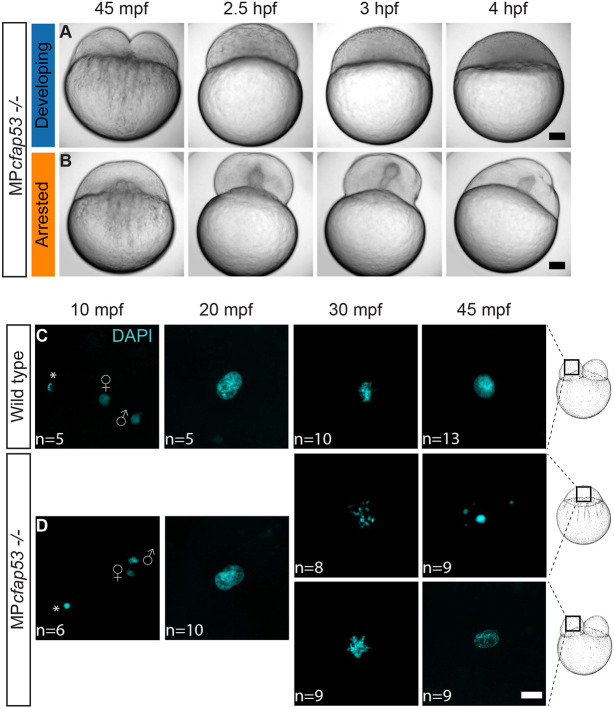
**MP*cfap53*^−/−^ embryos arrest and display aberrant nuclear morphology.** (A,B) Time series of maternal and paternal (MP)*cfap53*^−/−^ embryos from 45 mpf up to 4 hpf. Although a small fraction of the MP*cfap53*^−/−^ embryos displayed normal development (A, *n*=15), most embryos arrested at the one-cell stage (B, *n*=52). (C,D) Confocal images of wild-type (C) and MP*cfap53*^−/−^ (D) embryos at indicated stages of development stained with DAPI to visualize nuclei, indicating that aberrant nuclear morphology starts to form at 30 mpf. Results were obtained from two different *cfap53*^−/−^ pairs. Asterisks indicate polar bodies. Gender symbols indicate a male or female pro-nucleus. Scale bars: 100 μm in A,B; 20 μm in C,D. Schematics of embryonic development are adapted, with permission, from Kimmel et al. (1995).

**Fig. 2.**
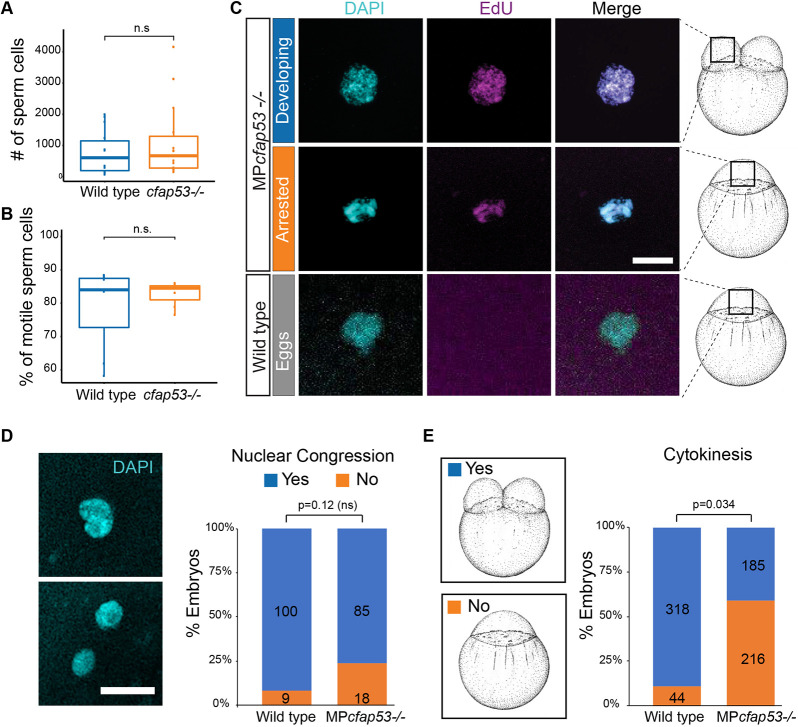
***cfap53*^−/−^ fish are fertile.** (A,B) Boxplots for sperm cell analysis showing the total number (horizontal lines) of sperm cells counted (A) and percentage of motile sperm cells (B) in spermatozoa derived from wild type (*n*=7) and *cfap53*^−/−^ males (*n*=7). Boxes indicate upper and lower quartiles and whiskers indicate upper and lower extremes. (C) Confocal images showing DAPI staining and EdU incorporation in MP*cfap53*^−/−^ embryos and lack of EdU incorporation in unfertilized eggs. EdU incubation was performed from 0 to 60 mpf. Quantification of the results are presented in Table 2. (D) Confocal images of one-cell stage embryos stained with DAPI showing either lack of nuclear congression (bottom) or initiation of nuclear congression (top) of male and female pronuclei. Graph shows a quantification of observed nuclear congression in wild-type and MP*cfap53*^−/−^ embryos. (E) Graph shows quantification of observed cytokinesis. Numbers of embryos analyzed are indicated in the bars. Embryos were obtained from at least three independent pairs. An unpaired Student's *t*-test was used to test for significance. Scale bars: 20 μm. Schematics of embryonic development are adapted, with permission, from Kimmel et al. (1995).

When homozygous *cfap53^−/−^* fish were crossed, we observed in independent crosses that 25-100% of the progeny did not develop into normal embryos but instead arrested at the first cell cleavage with a single nucleus (Table 1). In time-lapse movies of these embryos lacking maternal (M) and paternal (P) *cfap53* (hereafter referred to as MP*cfap53^−/−^*) we observed temporarily invaginations of the cell membrane, but these never resulted in cell divisions (Fig. 1A,B). As we could not distinguish the MP*cfap53*^−/−^ eggs before the first cell division we fixed MP*cfap53*^−/−^ eggs at different stages after fertilization and stained nuclear DNA with DAPI (Fig. 1C,D). Although at 10 min post fertilization (mpf) and 20 mpf no differences were apparent, we observed at 30 mpf and 45 mpf that ∼50% of MP*cfap53*^−/−^ embryos displayed an aberrant and variable nuclear morphology with dispersed chromosomes and micronuclei (Fig. 1C,D: 30 mpf: *n*=8/17; 45 mpf: *n*=9/18). Importantly, the MP*cfap53*^−/−^ embryos in which the first cell division did occur (Fig. 1A) developed normally and could be grown to viable and fertile adults. Interestingly, embryos derived from either homozygous *cfap53*^−/−^ males or females crossed to an otherwise wild-type female or male, respectively, displayed the same arrest in the first cell division (Table 1). As unfertilized eggs arrest in a similar manner, we investigated whether the arrested *cfap53*^−/−^ embryos were fertilized. We first analyzed sperm cell number and motility of *cfap53*^−/−^ males, and did not observe any significant differences with sperm isolated from wild-type males (Fig. 2A,B, Table S1). Next, we analyzed whether DNA replication was initiated in MP*cfap53*^−/−^ embryos, as this occurs only in fertilized eggs (Dekens et al., 2003). Indeed, we observed that EdU incorporation correlates well with fertilization as wild-type embryos incubated with EdU directly after fertilization and analyzed at 45 mpf (at the two-cell stage) efficiently incorporated EdU into their DNA (Fig. 2C and Table 2A; wild-type cross embryos at the two-cell stage, EdU^+^: *n*=23/23). This was in contrast to unfertilized (squeezed) eggs, as we never observed EdU incorporation in unfertilized eggs (Fig. 2C and Table 2A; unfertilized eggs, EdU^+^: *n*=0/23) as reported earlier (Dekens et al., 2003). In crosses from *cfap53*^−/−^ pairs, we found that 53% of the arrested embryos had not incorporated the EdU, indicating that these were not fertilized or did not take up the EdU efficiently (Table 2A; *cfap53*^−/−^ cross embryos at the one-cell stage, EdU^−^: *n*=29/55). Importantly, we observed that 47% of arrested MP*cfap53*^−/−^ embryos efficiently incorporate EdU (Fig. 2C and Table 2A; *cfap53*^−/−^ cross embryos at the one-cell stage, EdU^+^: *n*=26/55), demonstrating that these embryos, although being fertilized, failed to complete cytokinesis. As we observed the cell division arrest in embryos derived from either *cfap53*^−/−^ males crossed with wild-type females or *cfap53*^−/−^ females crossed with wild-type males, we also tested the EdU incorporation in such embryos. These embryos are referred to as P*cfap53*^−/−^ embryos (for lacking paternal Cfap53) and M*cfap53*^−/−^ embryos (for lacking maternal Cfap53). Importantly, EdU was incorporated in both P*cfap53*^−/−^ embryos and M*cfap53*^−/−^ embryos (Table 2a). The EdU incorporation was very efficient in P*cfap53*^−/−^ embryos (EdU^+^: *n*=14/16), which indicates that *cfap53*^−/−^ sperm are very effective in fertilizing eggs. This is consistent with the observed normal sperm numbers and their motility. The EdU incorporation was less efficient for the M*cfap53*^−/−^ embryos (EdU^+^: *n*=18/33), suggesting a lower fertilization rate.

**Table 1.**
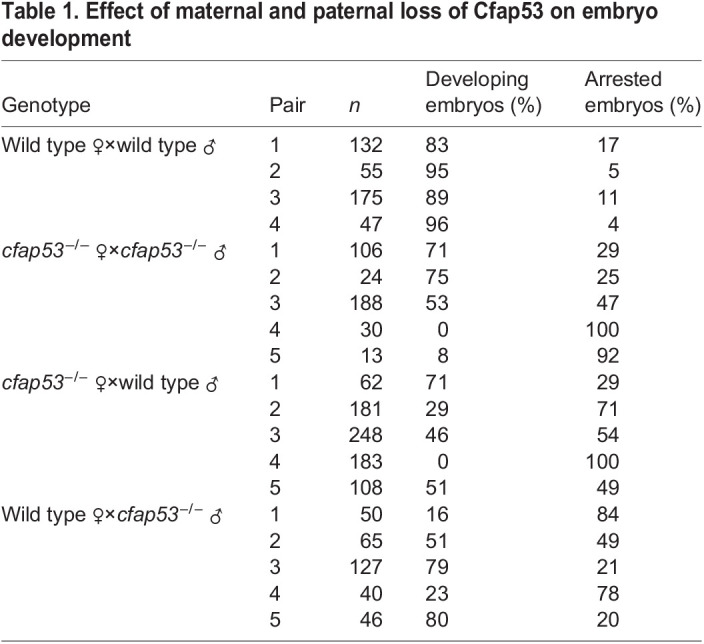
Effect of maternal and paternal loss of Cfap53 on embryo development

**Table 2.**
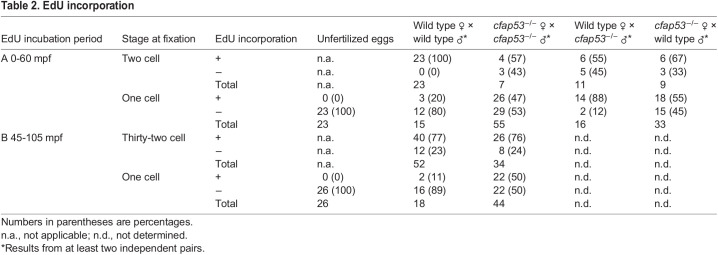
EdU incorporation

After fertilization, the male and female pronuclei come together during the nuclear congression, after which they fuse to form the zygotic nucleus. To analyze whether nuclear congression is affected in MP*cfap53*^−/−^ embryos, we took clutches of MP*cfap53*^−/−^ embryos and fixed some of the embryos at 15 mpf to analyze nuclear congression (Fig. 2D), while the rest of the clutch was analyzed live for cytokinesis (Fig. 2E). We analyzed at least three independent wild-type and *cfap53*^−/−^ pairs, and found no significant difference in nuclear congression between MP*cfap53*^−/−^ and wild-type embryos (Fig. 2D). Cytokinesis, however, was significantly less frequent in MP*cfap53*^−/−^ embryos (Fig. 2E). These results indicate that nuclear congression is not affected in MP*cfap53*^−/−^ embryos*.*

Next, we addressed whether DNA replication is continued in the in MP*cfap53*^−/−^ embryos in which cytokinesis failed, as was reported for *fue* mutants (Dekens et al., 2003). We therefore incubated embryos with EdU starting at 45 mpf and observed that 50% of the arrested MP*cfap53*^−/−^ embryos still incorporated EdU (Table 2B; *cfap53*^−/−^ cross embryos at the one-cell stage, EdU^+^: *n*=22/44). These results indicate that when cytokinesis fails in MP*cfap53*^−/−^ embryos, DNA replication can continue resulting in endoreduplication. In summary, although maternal- and paternal-provided Cfap53 is not required for nuclear congression, it facilitates karyokinesis and cytokinesis.

### Cfap53 interactome

**Fig. 3.**
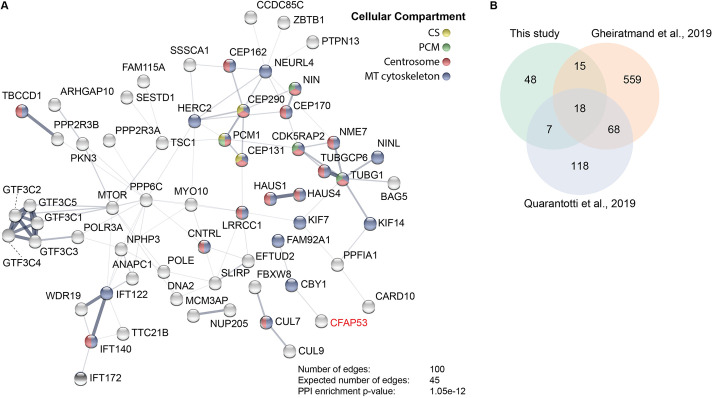
**Cfap53 interacts with centrosomal, centriolar satellite and microtubule-associated proteins.** (A) Network analysis using String-DB (Szklarczyk et al., 2015) from proteins identified by MS analysis after streptavidin-based purification using CFAP53 as bait. Only high-confidence hits from the MS dataset were used, which were obtained via stringent filtering steps on raw data (see Materials and Methods). Proteins shown are those with a significantly higher number of edges (representing protein-protein associations) than that expected stochastically. The thickness of the lines between the nodes does not represent strength or specificity of a given interaction, but represents the approximate confidence on a scale from zero to one of a found association being true, given the available evidence. Node colors represent cellular compartment of the indicated protein. (B) Venn diagram showing number of proteins identified in this study by bioGFP-Cfap53 pulldown with MS analysis overlapping with the centriolar satellite proteins that were identified by Gheiratmand et al. (2019) and Quarantotti et al. (2019).

Coiled-coil domain-containing proteins often act as scaffolds for large protein complexes, which is consistent with the observation that in cultured cells Cfap53 protein localizes to centriolar satellites and is required for their integrity (Silva et al., 2016). To identify proteins that interact with Cfap53, we performed Cfap53 protein pull downs followed by mass spectrometry (MS). Compared with cytoplasmic proteins, zebrafish embryos contain relatively large amounts of yolk proteins, which could hamper such an approach. We therefore expressed human CFPA53 fusion proteins [with N-terminal or C-terminal biotinylated GFP (bioGFP)] in HEK293 cells (Fig. S1). In HEK293 cells with low levels of BioGFP-CFAP53, the fusion protein localized to centrosomes, marked by γ-tubulin (Fig. S1). Streptavidin-based pull downs followed by MS resulted in the retrieval of in total 1148 proteins that were pulled down by both the bioGFP-CFAP53 and the CFAP53-bioGFP. For these two biological samples, the Pearson correlation of the MS scores across all proteins that were detected in both pull downs was 0.71, demonstrating high reliability of the data. Next, the MS scores from both experiments were combined and used for further statistical analysis using the significance analysis of interactome (SAINT) and fold change calculation (Choi et al., 2011; Mellacheruvu et al., 2013) (see detailed description in the Materials and Methods). This resulted in the identification of 88 proteins as putative binding partners of Cfap53 (Table S2). Network and GO analysis enabled us to group these proteins into functional classes (Fig. 3A, Fig. S2).

A large number of proteins from our list are known for their localization in centrosomes and centriolar satellites, such as PCM1, NIN, CEP131, CEP290 and CEP170, which is consistent with the observed colocalization of Cfap53 and PCM1 (Balczon et al., 1994; Kim et al., 2008; Kubo et al., 1999; Kubo and Tsukita, 2003; Quarantotti et al., 2019; Silva et al., 2016; Staples et al., 2012; Stowe et al., 2012). PCM1 acts as a scaffold for centriolar satellite assembly and facilitates centrosomal protein trafficking (Balczon et al., 1994; Kubo et al., 1999; Kubo and Tsukita, 2003). We therefore compared our protein list with a published list of 211 satellite proteins resulting from an affinity purification of satellites using PCM1 as bait (Gheiratmand et al., 2019; Quarantotti et al., 2019). We indeed observed a significant overlap with these interactomes, as 25 out of 88 proteins (28%, *P*=1.8×10^−22^) that were enriched in the bioGFP-Cfap53 pull down were also present in the PCM1 affinity purification (Fig. 3B). In addition, a proximity-dependent biotin identification approach with 22 satellite proteins, including Cfap53, identified an interactome of 660 proteins (Gheiratmand et al., 2019). Comparison with the Cfap53 interactome revealed that 33 out of 88 proteins (37%, *P*=6.4×10^−18^) overlapped with the centrosomal satellite proteome (Fig. 3B).

Another prominent group of proteins copurifying with Cfap53 are the γTuRC components γ-tubulin (TUBG1) and GPC6, as well as proteins that interact with γTuRC, such as CDK5RAP2 and the kinase NME7 (Choi et al., 2010; Liu et al., 2014; Murphy et al., 2001). Moreover, the MS data contained components of HAUS complex (HAUS1 and HAUS4), which localizes to spindles and centrosomes, and controls branching microtubule nucleation (Goshima et al., 2008; Lawo et al., 2009). Together, these data show that Cfap53 can interact with centrosomal and centriolar satellite proteins, as well as proteins with known functions in microtubule nucleation.

### Cfap53 localizes to MTOC during embryonic development

**Fig. 4.**
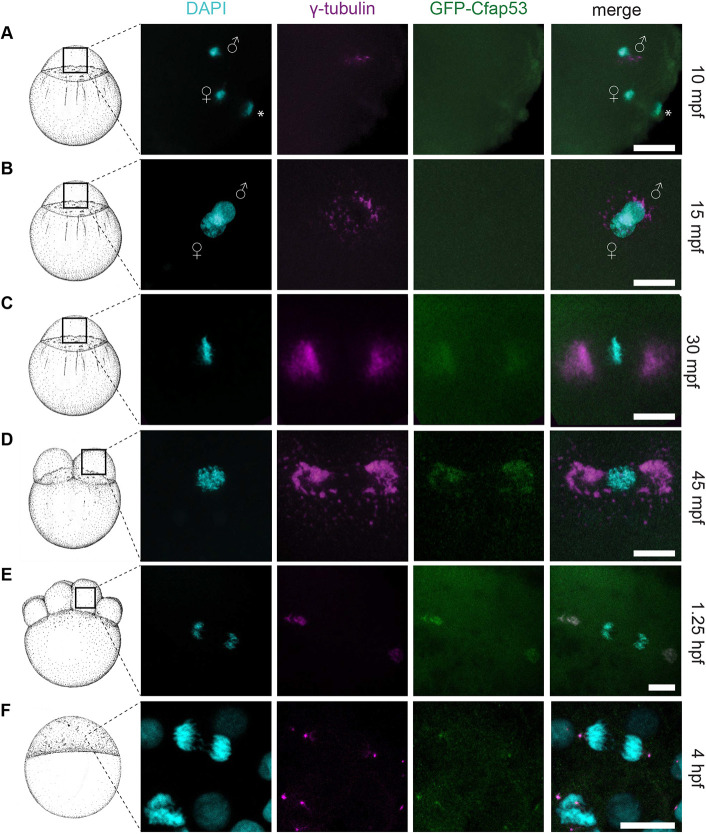
**GFP-Cfap53 localization during cleavage stages.** (A-F) Maximal projections of confocal stacks from Tg(*ubi:GFP-cfap53*) embryos immunolabelled for GFP, γ-tubulin and DAPI from 30 mpf until 4 hpf. (A) At 10 mpf, male and female pronuclei and the female polar body are visible. Some γ-tubulin foci around the male pronucleus can been observed, while GFP-Cfap53 has a diffuse localization (A, *n*=7). (B) At 15 mpf, both pronuclei are almost fused and some γ-tubulin can be observed around the nuclei, while GFP-Cfap53 localization is still diffuse (B, *n*=7). (C) At 30 mpf, chromosomes have aligned along the metaphase plate and strong γ-tubulin staining can be observed on either side of it. At this moment, GFP-Cfap53 also starts to localize to both sides of the metaphase plate, where it colocalizes with γ-tubulin (C, *n*=12). (D) At 45 mpf, the first cell division has completed and one of the two nuclei is shown. Both GFP-Cfap53 and γ-tubulin are visible in broad domains on both sides of the nucleus during prophase (D, *n*=8). (E) Single cell of an eight-cell stage blastula with segregating chromosomes during telophase of mitosis. GFP-Cfap53 and γ-tubulin colocalize in a more condensed region at both sites of the division plane (E, *n*=7). (F) Blastula cell in telophase during mitosis at the sphere stage (4 hpf). GFP-Cfap53 and γ-tubulin colocalize to a confined region on both sides of the division plane (F, *n*=1). Asterisks indicate polar bodies. Gender symbols indicate male or female pro-nucleus. Scale bars: 20 μm. Schematics of embryonic development are adapted, with permission, from Kimmel et al. (1995).

As Cfap53 localization in zebrafish embryos had not been determined, we generated a Tg(*ubi:GFP-Cfap53*) line, in which a GFP-Cfap53 fusion protein is expressed ubiquitously. To test whether the GFP-Cfap53 protein is functional, the Tg(*ubi:GFP-Cfap53*) was crossed into the *cfap53^hu1047^* line. We first incrossed Tg(*ubi:GFP-Cfap53*)/*cfap53^+/−^* fish and analyzed their progeny for laterality defects, as we previously described laterality defects in *cfap53*^−/−^ embryos (Noël et al., 2016). We found that the laterality defect was rescued in *cfap53*^−/−^ embryos expressing the GFP-Cfap53 fusion protein (Fig. S3). Next, we crossed adult *cfap53*^−/−^ fish that carried the Tg(*ubi:GFP-Cfap53*), and their progeny were scored for GFP-Cfap53 expression and embryonic development. Importantly, GFP-Cfap53 was also able to rescue the maternal *cfap53*^−/−^ phenotype, as the percentage of arrested embryos in MP*cfap53*^−/−^ embryos dropped from 67% to 20% when maternal GFP-Cfap53 was present (Fig. S3). From these results, we concluded that GFP-Cfap53 is a functional protein and therefore could be used to determine the subcellular localization of Cfap53. We investigated the subcellular localization of GFP-Cfap53 in embryos starting at 10 mpf up to 4 hpf and compared it with γ-tubulin localization as a marker for the MTOC (Lindeman and Pelegri, 2012; Rathbun et al., 2020; Yabe et al., 2007). During the first 15 min of development, we observed a weak GFP-Cfap53 signal equally distributed throughout the cytoplasm while some γ-tubulin was localized around the male pronucleus (Fig. 4A,B). At 30 mpf, when the first mitosis of the zygote has started, we observed accumulation of GFP-Cfap53 on either side of the metaphase plate containing the aligned chromosomes. At this stage, we observed for the first time a colocalization of GFP-Cfap53 with γ-tubulin (Fig. 4C). As embryo development proceeds with a new cell division every 15 min, both GFP-Cfap53 and γ-tubulin localization became more confined to two structures on either side of the aligned or segregating chromosomes (Fig. 4E,F). Together, these results indicate that, in the egg, GFP-Cfap53 is initially diffusely localized throughout the cytoplasm and that after pronuclear fusion both Cfap53 and γ-tubulin accumulate to form large MTOCs consisting of multiple protein foci. As we observed that paternal Cfap53 facilitates the initiation of the first cell division, we investigated GFP-Cfap53 localization in sperm cells. We observed that the GFP-Cfap53 localizes near the sperm DNA in two domains. The first domain is around the centrioles marked by centrin and the second domain is in a single spot of unknown origin near the centrioles (Fig. S4).

### Cfap53 facilitates the formation of the MTOC after fertilization

**Fig. 5.**
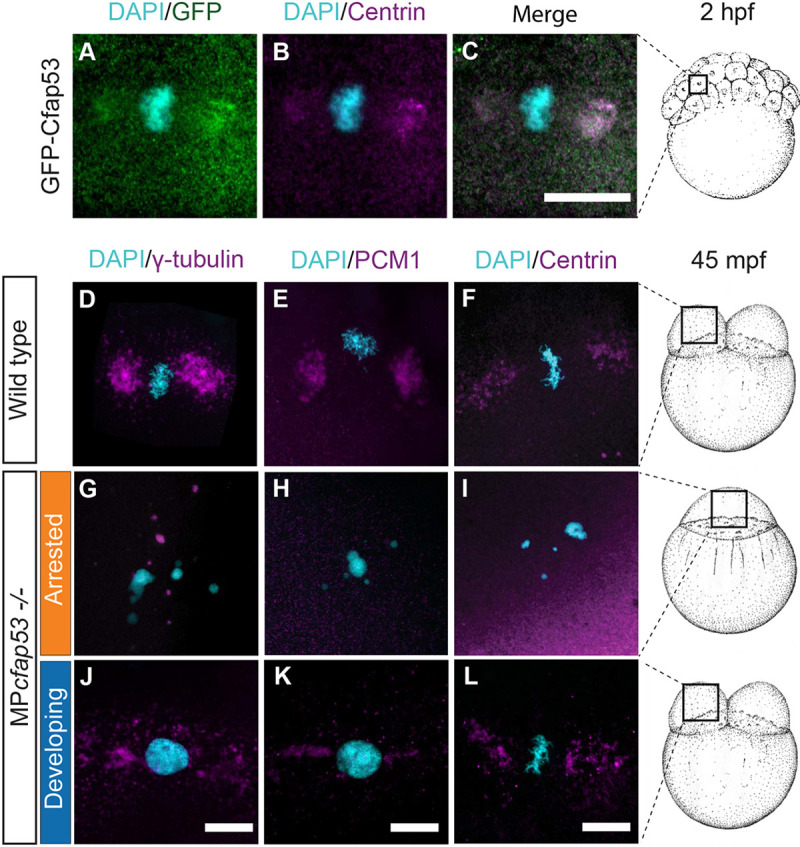
**Cfap53 is important for the localization of centrosomal components and centriolar satellites at the MTOC.** (A-C, *n*=9) Maximal projections of confocal stacks from fixed Tg(ubi:*GFP-cfap53*) transgenic embryos immunolabeled for centrin at 2 hpf shows strong colocalization of GFP-Cfap53 with centrin during metaphase. (D-L) Maximal projections of confocal stacks from fixed wild-type or MP*cfap53*^−/−^ embryos at 45 mpf immunolabeled for centrosomal components. γ-tubulin (D; *n*=8; G, *n*=15; J, *n*=3), PCM1 (E, *n*=7; H, *n*=15; K, *n*=3) and centrin (F, *n*=5; I, *n*=8; L, *n*=14) in wild-type embryos (D-F), MP*cfap53*^−/−^ non-developing embryos (G-I) or MP*cfap53*^−/−^ developing embryos (J-L) at 45 mpf fixed. Scale bars: 20 μm. Schematics of embryonic development are adapted, with permission, from Kimmel et al. (1995).

To confirm that GFP-Cfap53 localizes to the MTOC in the zebrafish embryo, we co-stained GFP-Cfap53-expressing embryos with antibodies that recognize centrin. Indeed, we observed colocalization of GFP-Cfap53 with centrin (Fig. 5A-C).

Based on the Cfap53 pull down results and its localization, we examined a possible role of Cfap53 in the formation of the MTOC. As the MS data revealed a possible interaction between Cfap53 with PCM1 and γ-tubulin, we investigated the localization of these and other centrosomal proteins in wild-type and MP*cfap53*^−/−^ embryos. In wild-type embryos, γ-tubulin, PCM1 and centrin were localized in the large MTOC structures typical for early cleavage stage zebrafish embryos (Fig. 5D-F) (Dekens et al., 2003; Lindeman and Pelegri, 2012; Rathbun et al., 2020). In arrested MP*cfap53*^−/−^ embryos, γ-tubulin signal was present, albeit at reduced levels, and it formed granule-like foci scattered throughout the cytoplasm (Fig. 5G). The mislocalization of γ-tubulin observed in MP*cfap53*^−/−^ embryos was rescued by expression of GFP-Cfap53, resulting in normal chromosome segregation (Fig. S5). In addition, PCM1 and centrin signals were diffuse and no longer accumulated in MTOC structures in arrested MP*cfap53*^−/−^ embryos (Fig. 5H,I). In developing MP*cfap53*^−/−^ embryos, we observed that γ-tubulin, PCM1 and centrin accumulated at the MTOCs with slight variations in their patterns (Fig. 5J-L), which may be due to natural variation observed among embryos.

**Fig. 6.**
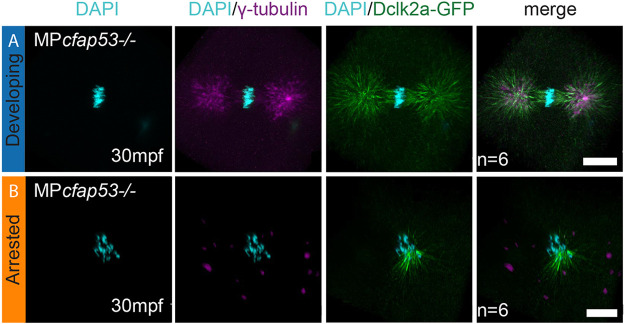
**Cfap53 facilitates assembly of the zygotic spindle.** (A,B) Maximal projections of confocal stacks on Tg*(XlEef1a1:dclk2a-GFP)*/MP*cfap53*^−/−^ embryos at 30 mpf and immunolabeled for GFP, γ-tubulin and DAPI. While six embryos formed centrosomes and a normal mitotic spindle during the first zygotic cell division (A, *n*=6), six embryos failed to assemble the centrosomes and the mitotic spindle (B, *n*=6). Instead, microtubule originating from the chromosomes is observed in these embryos. Scale bars: 20 μm.

Failure in MTOC formation results in a disorganized microtubule network and cell cycle arrest, as proper bipolar spindles cannot be formed (Minshull et al., 1994). To test whether the observed arrest in the first cell cycle of MP*cfap53*^−/−^ embryos could be due to defective bipolar spindle formation, we used the Tg(*XlEef1a1:dclk2a-GFP*) line, which labels microtubules *in vivo*. As the Dclk2a-GFP fusion protein is maternally provided, we used it to visualize the microtubule network during the first cell division. In developing MP*cfap53* embryos, we observed microtubule bundles nucleating from the MTOC to form astral microtubules and a symmetric bipolar spindle (Fig. 6A). However, in MP*cfap53*^−/−^ arrested embryos the bipolar spindle was not formed; instead, we observed that microtubule bundles still nucleate but in a disorganized fashion originating from the chromosomes (Fig. 6B), which explains their cell cycle arrest. Taken together, these results indicate that Cfap53 facilitates MTOC formation and therefore the assembly of the mitotic spindle.

### The microtubule network is important for the zygotic MTOC

**Fig. 7.**
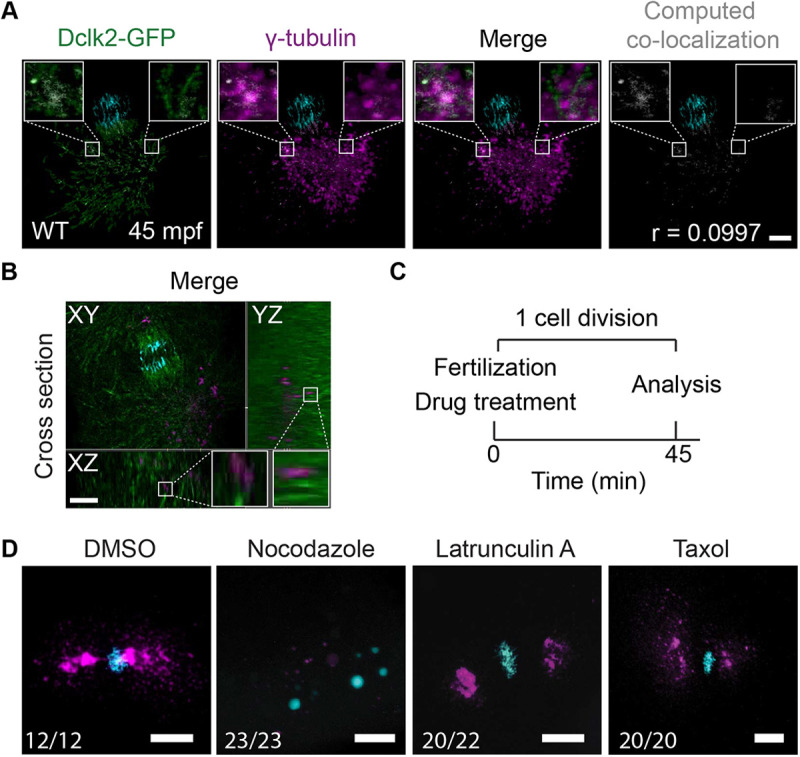
**Microtubule-dependent γ-tubulin localization.** (A,B) Maximal projections of confocal stacks from a Tg*(Xla.Eef1a1:Dclk2a-GFP)* embryo immunolabeled for DAPI (in cyan), Dclk2a-GFP (in green) and γ-tubulin (in magenta) at 45 mpf fixed during telophase. Colocalization of γ-tubulin and Dclk2a-GFP is shown in white in the merge. Computation of colocalization gives an average Pearson's coefficient of 0.1, indicating that there is very little colocalization of γ-tubulin and Dclk2a-GFP (A, *n*=5). Scale bar: 100 μm. (B) Optical cross-section through 45 mpf embryo immunostained for γ-tubulin and Dclk2a-GFP at the region of one of the centrosomes, which shows that γ-tubulin is localized directly adjacent to the microtubule bundles (B, *n*=5). Scale bar: 100 μm. White squares outline the areas shown in more detail. (C) Schematic overview of the drug treatment experiment (C), for which the results are shown in D. (D) Maximal projections of confocal stacks of embryos at 45 mpf immunostained with γ-tubulin (in magenta) and DAPI (in cyan) fixed during telophase. Nocodazole treatment (*n*=23) prevents γ-tubulin accumulation at the MTOC. Lantrunculin A (*n*=20) and taxol (*n*=20) treatments have no effect on γ-tubulin localization to the MTOC. Scale bars: 20 μm.

When analyzing the Dclk2a-GFP and γ-tubulin colocalization more closely, we noticed that γ-tubulin forms foci varying in size at apparent microtubule ends or dispersed along microtubules. The Dclk2a-GFP and γ-tubulin did not appear to colocalize (Fig. 7A,B), which was confirmed by a very low Pearson's coefficient of 0.0997 for Dclk2a-GFP and γ-tubulin colocalization (Fig. 7B). This observation suggests that at this stage γ-tubulin is associated with the microtubules, similar to what has been shown for PCM1 (Kubo and Tsukita, 2003). Therefore, we investigated whether microtubules are required for γ-tubulin localization at the MTOC. We treated wild-type embryos directly after their fertilization with nocodazole or taxol to either depolymerize or stabilize microtubules, respectively (Fig. 7C). Although taxol treatment arrested the embryos at the metaphase stage during the first mitosis, there was no strong effect on γ-tubulin localization (Fig. 7D). Interestingly, embryos treated with nocodazole showed dispersed cytoplasmic granules of γ-tubulin, similar to those seen in MP*cfap53*^−/−^ embryos (compare Fig. 7D with Figs 5D and 6B). We therefore conclude that, in the zebrafish embryo, a functional microtubule network is required for the localization of γ-tubulin at the MTOC.

## DISCUSSION

After fertilization, a MTOC is assembled from maternal and paternal components. Although this is a crucial step to ensure formation of the sperm aster and the mitotic spindle, which facilitates proper cell division and further development of the embryo, very little is known about its mechanisms and the factors that are involved. In this study, we describe that both maternally and paternally derived Cfap53 protein plays a specific role in the MTOC to form the mitotic spindle, although its role is dispensable for sperm aster assembly, as assessed by normal pronuclear congression.

The cytokinesis defect that we observed in MP*cfap53*^−/−^ embryos has an incomplete penetrance (see Table 1). This suggests that the loss of Cfap53 can be compensated for by other mechanisms (see below discussion about diffusion versus active protein transport) or by proteins with a similar function. Recent genetic work in zebrafish has shown that mutations that introduce a premature stop codon in the mRNA, which results in mRNA degradation, can trigger a transcriptional adaptation mechanism (Rossi et al., 2015; Sztal et al., 2018; Zhu et al., 2017). This transcriptional adaptation mechanism causes an upregulation of the expression of related genes and thereby (partially) rescues the phenotype. As the *cfap53^hu10478^* allele is a 7 bp deletion, it results in a frameshift and subsequent introduction of a premature stop codon, which may result in mRNA degradation and activation of the transcriptional adaptation mechanism. mRNA sequencing of oocytes from *cfap53* mutant fish could help to identify the compensating genes.

Our genetic analysis indicates that both maternally and paternally provided Cfap53 protein is important for progression through the first cell division (Fig. 1). Among the very few genes that have been associated with both maternal and paternal functions during the first cell division in *C. elegans* and zebrafish is *zyg-1/sass6* (Yabe et al., 2007; O'Connell et al., 2001). Our finding that paternal Cfap53 is important is consistent with the detection of Cfap53 protein in bovine sperm by mass spectrometry (Firat-Karalar et al., 2014) and our own results demonstrating the presence of GFP-Cfap53 protein in zebrafish sperm by microscopy (Fig. S4). Thus, although the sperm cell loses most of its PCM and centriolar satellite proteins, Cfap53 is maintained during spermatogenesis. The location of GFP-Cfap53 in sperm is atypical, as most of the protein does not colocalize with the centriole marker. Future research should address the mechanism by which Cfpa53 from the sperm contributes to the formation of the mitotic spindle.

After the sperm aster is formed and nuclear congression is completed, GFP-Cfap53 localizes to either side of the fused maternal and paternal pronuclei. This coincides with a clear γ-tubulin accumulation on either side of the nucleus. In earlier stages, we observed only a diffuse GFP-Cfap53 signal, which either indicates a lack of specific localization or a lag in the translation of GFP-Cfap53 protein after fertilization. Lack of Cfpa53 accumulation at the sperm aster is consistent with unaffected nuclear congression in MPcfap53^−/−^ embryos. This is very different from the role of lymphoid-restricted membrane protein (Lrmp), a maternally provided protein required for nuclear congression (Dekens et al., 2003; Lindeman and Pelegri, 2012). The different roles of Cfap53 in the MTOCs forming the sperm aster and the mitotic spindle may be related their difference in composition and size. We and others have observed that PCM1, centrin and γ-tubulin form cytoplasmic accumulations that appear as a large clouds on either side of the nucleus during the first cell divisions of the zebrafish embryo (Dekens et al., 2003; Lindeman and Pelegri, 2012; Rathbun et al., 2020; Stowe et al., 2012). Large MTOCs have also been observed in one-cell stage *Xenopus* embryos and embryos from marine invertebrates (Mitchison, et al., 2012). Why these structures that function as MTOCs are so large at the one-cell stage and become smaller during subsequent cell divisions is not clear, but may be related to the large size of the embryo (see also below). In *C. elegans* embryos, the MTOC does indeed scale with cell size, which is regulated by limited maternal protein pools (Decker et al., 2011). PCM1-, centrin- and γ-tubulin-containing structures depend on Cfap53, as these were not formed in MP*cfap53* embryos. The biochemical function of Cfap53 is currently unclear. It has three predicted coiled-coil domains, which are low-complexity structures that facilitate protein-protein interactions and are found in numerous centriolar satellite proteins (Gheiratmand et al., 2019; Mason and Arndt, 2004; Quarantotti et al., 2019). It is possible that, similar to the coiled-coil proteins SPD-5, which is a *C. elegans* centrosome component, CFAP53 facilitates MTOC formation by passive liquid-liquid phase separation (Woodruff, 2018; Woodruff et al., 2017).

What could be the mechanism by which Cfap53 recruits γ-tubulin and other components towards the MTOC after fertilization? In general, protein targeting to the MTOC is regulated by two different pathways: (1) active microtubule-based transport using centriolar satellites; and (2) concentration of proteins to the MTOC via passive diffusion (Conkar et al., 2019; Gillingham and Munro, 2000; Hori and Toda, 2017; Khodjakov and Rieder, 1999; Young et al., 2000; Zimmerman and Doxsey, 2000). For a subset of centrosomal proteins, passive diffusion is sufficient in somatic cells, which includes γ-tubulin (Khodjakov and Rieder, 1999). However, passive diffusion of γ-tubulin alone may not be sufficient, as zebrafish embryos have a much larger volumes during the first cell divisions compared with average somatic cells (1000 times larger in case of the zebrafish). This can be overcome by active directional microtubule- and motor-dependent transport. Several studies have shown that recruitment of γ-tubulin towards the centrosome in somatic cells is facilitated by active transport that is dependent on the microtubule network (Quintyne et al., 1999; Schnackenberg et al., 1998; Stearns and Kirschner, 1994; Young et al., 2000). Our observation that γ-tubulin-positive clusters align along microtubules (Fig. 7A,B) and our finding that nocodazole treatment of wild-type zebrafish embryos directly after fertilization inhibits MTOC localization of γ-tubulin (Fig. 7C) are consistent with microtubule dependency of γ-tubulin recruitment.

How is Cfap53 facilitating the active localization of γ-tubulin at the MTOC? Based on its localization in somatic cells, CFAP53 was classified as a centriolar satellite protein (Silva et al., 2016). Both our MS analysis in somatic cells and the widespread granular localization of GFP-Cfap53 in two-cell stage zebrafish embryos, which resembles localization of the satellite marker PCM1, is consistent with localization of Cfap53 in centriolar satellites (Figs 3 and 4D, Table S2). Centriolar satellites are membraneless granules containing proteins that associate and move along microtubules in a dynein-dependent manner to deliver proteins to the centrosome (Dammermann and Merdes, 2002; Kubo et al., 1999). We find that γ-tubulin is highly represented in our GFP-Cfap53 MS analysis, and we observed the simultaneous accumulation and strong colocalization of GFP-Cfap53 with γ-tubulin at the two-cell stage (Figs 3A and 4D, Table S2), which would suggest a direct role for Cfap53 in γ-tubulin localization to the MTOCs. Alternatively, Cfap53 has a more indirect role in facilitating γ-tubulin localization to the MTOCs. We found NME7 and CDK5RAP2 in our MS data, which are both part of the γ-tubulin ring complex and are recruited in a dynein-dependent manner (Fig. 3A, Table S2) (Choi et al., 2010; Hutchins et al., 2010; Jia et al., 2013; Liu et al., 2014). Defects in centriolar satellites from MP*cfap53* mutant embryos may therefore result in the absence of other proteins that are required to recruit γ-tubulin. Importantly, the loss of centriolar satellites in somatic cells, by PCM1 depletion, has little effect on the integrity of the centrosome (Gheiratmand et al., 2019), indicating a clear difference in the role of centriolar satellites in fertilized eggs and somatic cells.

In conclusion, we discovered a novel function for paternally and maternally derived Cfap53 in the formation of the mitotic spindle in zebrafish embryos. Although Cfap53 protein is dispensable for the function of the sperm aster during nuclear congression, it aids the formation of the MTOC from which the mitotic spindle is formed.

## MATERIALS AND METHODS

### Zebrafish genetics and strains

The following fish lines were used in this study: Tupfel Longfin (TL), *cfap53^hu10478^* (Noël et al., 2016), Tg(*h2afz:GFP*) (Pauls et al., 2001), Tg(*Xla.Eef1a1:dclk2a-GFP*) (Tran et al., 2012) and Tg(*ubi:GFP-cfap53*). Fish were maintained at 27.5°C in a 14/10 h light/dark cycle, according to standard laboratory conditions (Aleström et al., 2020). Embryos were collected and staged according to Kimmel et al. (1995). Animal experiments were approved by the Animal Experimentation Committee (DEC) of the Royal Netherlands Academy of Arts and Sciences (KNAW).

### Plasmid construction and transgenesis

The following plasmids were generated in this study: bioEGFP-CFAP53, CFAP53-EGFPbio and GFP-Cfap53 containing tol2 sites. Human *CFAP53* coding sequence (CDS) was obtained by performing PCR on a PCS2 construct containing human *CFAP53* cDNA and cloned into the pbioEGFP-C1 and pbioEGFP-N1 constructs using the Gibson cloning system (Noël et al., 2016), which are modified pEGFP-C1 and pEGFP-N1, respectively, in which a linker encoding the sequence MASGLNDIFEAQKIEWHEGGG (which is the substrate of biotin ligase BirA) was inserted in front of or behind the eGFP-encoding sequence.

The zebrafish *GFP-cfap53* construct was obtained from Narasimhan et al. (2015) and cloned into the pME-MCS followed by recombination with p5E′-Ubi (Mosimann et al., 2011), p3E-polyA and pDestTol2pA7 using the multisite gateway cloning strategy (Kwan et al., 2007). The plasmid DNA was injected at 30 ng/µl in the presence of 25 ng Tol2 mRNA for genomic integration and to generate Tg(*ubi:GFP-cfap53*). At 3 dpf, healthy embryos displaying robust GFP fluorescence in the cilia were selected and grown to adulthood. Subsequently, founder fish were identified by outcrossing and their progeny grown to adulthood to establish the transgenic line.

### EdU labeling

For EdU detection, the Click-iT EdU Imaging Kit with Alexa Fluor 488 (Thermo Fisher) was used. The stock solutions were prepared according to the provided protocol included in the kit (5 mg/ml EdU stock solution). For EdU incubation, a fresh working solution of 10% DMSO/50 µg/ml EdU diluted in E3 water was prepared in 1.5 ml Eppendorf tubes containing a total volume of 1 ml. Fish were set-up in pairs and eggs were collected either directly after laying or after 45 mpf. After collection the eggs were transferred into the EdU solution using a transfer pipet and incubated for 30 min on ice in the dark. The embryos were then transferred to a 28°C incubator to incubate for 30 min. After incubation, the embryos were transferred into fresh E3 water for a short rinse and directly fixed after with microtubule fix (3.7% formaldehyde, 0.25% glutaraldehyde, 5 mM EGTA and 0.3% Triton X-100) for 2 h at room temperature as described below in the ‘Fixation and antibody labeling’ section. Detection of EdU was performed according to the protocol described in the Click-iT EdU Imaging Kit. The imaging of the EdU detection was performed as described in the ‘Imaging’ section below.

### Cell culture

Human embryonic kidney 239T (HEK293T) and HeLA (Kyoto) cell lines were cultured in medium that consisted of 45% DMEM, 45% Ham's F10 and 10% fetal calf serum supplemented with penicillin and streptomycin. The cell lines were routinely checked for mycoplasma contamination using LT07-518 Mycoalert assay (Lonza). For the immunolabeling experiment, HeLa cells were transfected with plasmids using FuGENE 6 (Promega). For streptavidin pull-down assays from HEK293T cells, plasmids were transfected using polyethylenimine (Polysciences).

### Streptavidin pull-down assays

Streptavidin pull-down assays were performed from HEK293T cell lysates by co-expressing biotin ligase BirA with GFP-tagged constructs containing a biotinylation site (bioGFP-CFAP53 and CFAP53-GFPbio) and only expressing biotin ligase BirA as a negative control. Constructs were transfected together into HEK293 cells using polyethylenimine (PEI, Polysciences) with a 24 h incubation time for protein expression. M-280 Streptavidin Dynabeads (Invitrogen) were blocked in a buffer containing 20 mM Tris (pH 7.5), 20% glycerol, 150 mM NaCl and 10 µg chicken egg albumin followed by three washes with wash buffer containing 20 mM Tris (pH 7.5), 150 mM NaCl and 0.1% Triton X-100. HEK293T cells were collected in ice-cold PBS followed by lysis on ice in a buffer containing 20 mM Tris (pH 7.5), 150 mM NaCl, 1 mM MgCl2, 1% Triton X-100 and complete protease inhibitor cocktail (Roche). To separate cell debris, the lysates were cleared by centrifugation at 16,000 ***g*** at 4°C for 20 min. Cell lysates were incubated with pre-blocked streptavidin beads for 120 min at 4°C followed by five washes with a buffer containing 20 mM Tris (pH 7.5), 150 mM NaCl and 0.1% Triton X-100 (Hooikaas et al., 2019).

### Mass spectrometry analysis

Beads were re-suspended in 20 µl of Laemmli Sample buffer (BioRad) and supernatants were loaded on a 4-12% gradient Criterion XT Bis-Tris precast gel (BioRad). The gel was fixed with 40% methanol/10% acetic acid and then stained for 1 h using colloidal Coomassie dye G-250 (Gel Code Blue Stain Reagent, ThermoFisher Scientific). After in-gel digestion (Noordstra et al., 2016), all samples were analyzed on a Orbitrap Q-Exactive HF mass spectrometer (ThermoFisher Scientific) coupled to an Agilent 1290 Infinity LC. Peptides were loaded onto a trap column (Reprosil C18, 3 µm, 2 cm×100 µm; Dr. Maisch) with solvent A (0.1% formic acid in water) at a maximum pressure of 800 bar and chromatographically separated over the analytical column (Zorbax SB-C18, 1.8 µm, 40 cm×50 µm; Agilent) using 90 min linear gradient from 7-30% solvent B (0.1% formic acid in acetonitrile) at a flow rate of 150 nl/min. The mass spectrometer was used in a data-dependent mode, which automatically switched between MS and MS/MS. After a survey scan from 350-1500 m/z the 12 most abundant peptides were subjected to HCD fragmentation. For data analysis, raw files were processed using Proteome Discoverer 1.4 (Thermo Scientific). Database searches were performed using Mascot as search engine on the Human Uniprot database. Carbamidomethylation of cysteines was set as a fixed modification and oxidation of methionine was set as a variable modification. Trypsin was set as cleavage specificity, allowing a maximum of two missed cleavages. Data filtering was performed using percolator, resulting in 1% false discovery rate (FDR). Additional filters were search engine rank 1 and mascot ion score >20. To retrieve significant putative binding partners of CFAP53 additional analyses on the raw MS data were performed using contaminant repository for affinity purification (the CRAPome) implementing fold change scoring (FC) scoring and probalistic scoring using SAINT. The FC is calculated based on the ratio of average normalized spectral counts in bait purifications to negative controls. SAINT uses statistical modeling which provides a probability of true interaction (Choi et al., 2011; Mellacheruvu et al., 2013). The FC scoring is subdivided into a FCA (primary score based on BirA negative control pull down) and FCB (secondary score based on existing GFP negative control). The FCA and FCB cut-offs were set to seven and three, respectively. The SAINT probability cut-off was set to 0.95.

### Drug treatments

To destabilize or stabilize the microtubule network 4 μg/ml nocodazole (1 mg/ml stock dissolved in DMSO) and 10 μM taxol (1 mM stock dissolved in DMSO) were used, respectively. To inhibit actin, 25 μM latrunculin A (5 mM stock dissolved in DMSO) was used. Embryos from the same fish were collected directly after fertilization and treated with the drug or DMSO control for 45 min, corresponding to the duration of the first cell division. Embryos were subsequently washed in E3 medium prior to fixation in microtubule fix as below and stained for γ-tubulin.

### mRNA transcription and injection

The PCS2-GFP-Cfap53 construct was linearized with NotI and used as template for transcription of capped mRNA (mMessage SP6 kit, Ambion, AM1340). mRNA was purified using lithium chloride precipitation and re-suspension in nuclease-free water. mRNA was injected into the yolk of one-cell stage embryos in a volume of 1 nl at a concentration of 100 ng/µl.

### Fixation and antibody labeling

For immunolabeling, HeLa cells were fixed in −20°C methanol for 10 min. Cells were then permeabilized with 0.15% Triton X-100 in PBS for 2 min; subsequent wash steps were performed in PBS supplemented with 0.05% Tween-20. Epitope blocking and antibody labeling steps were performed in PBS supplemented with 0.05% Tween-20 and 1% BSA. Before mounting in Vectashield mounting medium (Vector Laboratories), slides were washed with 70% and 100% ethanol and air-dried (Hooikaas et al., 2019).

Zebrafish embryos were collected at the appropriate stages during development and fixed for 2 h at room temperature in microtubule fix (3.7% formaldehyde, 0.25% glutaraldehyde, 5 mM EGTA and 0.3% Triton X-100) at the indicated stages. After fixation, embryos were washed three times with PBS and Triton X-100 (0.3% PBST) and manually dechorionated. Embryos were dehydrated in methanol overnight at −20°C and rehydrated in a methanol:PBS series. Before labeling, embryos were treated with 0.5 mg/ml sodium borohydrate for 30 min at room temperature to inactivate remaining glutaraldehyde. Subsequently, embryos were washed in PBST and yolks were manually separated from the cells using forceps. Embryos were blocked in 0.3% PBST, 2% BSA and 10% sheep serum for 1 h at room temperature. Commercial antibodies used were as followed: anti-PCM1 (Sigma, rabbit polyclonal, 1:200), anti-γ-tubulin (Sigma, mouse monoclonal GTU-88, 1:500), anti-centrin (Millipore, monoclonal mouse IgG2aκ 1:200) and anti-GFP (Aves, chicken, 1:500). All primary antibodies were incubated overnight at 4°C. Primary antibodies were detected using Alexa Fluor 488 (Invitrogen, goat anti-mouse), Alexa Fluor 488 (Invitrogen, goat anti-chicken, IgG1) and Alexa Fluor 555 (Invitrogen, goat anti-mouse, IgG1). Nuclei were shown by DAPI (4′,6-diamidino-2-phenylindole) staining.

### Imaging

Fixed embryos were embedded in 0.25% agarose to be mounted on a glass bottomed dish. Subsequently, embryos were imaged using a Leica SP5 Multi Photon setup (Leica Microsystems) using a 25× water and 63× glycerol objective followed by a *z*-stack maximum projection (step size 1 µm).

For generation of time lapse videos, live embryos were embedded in 0.25% agarose to be mounted on a glass bottom dish. Subsequently, embryos were imaged live using a temperature controlled AF7000 Widefield Fluorescence Microscope setup (Leica Microsystems) using a 10× dry objective maintaining the temperature at a constant 28°C. The interval between images was set to 2 min. Computation of colocalization was performed using the 3D colocalization tool in Bitplane Imaris software (V9.3.1).

### Sperm motility evaluation by computer-assisted sperm analysis (CASA)

Sperm cells were extracted from *cfap53*^−/−^ and wild-type males as previously described (Dooley et al., 2013). Directly after collection, sperm cells were transferred into 50 μl of buffered sperm-motility inhibiting solution (BSMIS) on ice. Motility was assessed on the computer-assisted semen analysis (CASA) system Sperm Vision (version 3.5, Minitüb). The system was operated at room temperature with an automated stage and four-chamber slides of 20 µm depth (Leja). After sperm activation in tap water, a 3.0 µl aliquot was immediately transferred to each chamber. Motility assessments started ∼12 s after motility activation. Spermatozoa were examined at 200× magnification by a camera with a resolution of 648×484 pixels (Pulnix TM-6760CL, JAI A/S). The Sperm Vision software analyzed 15 successive fields in the central part of the chamber at a frame rate of 60 Hz per field. Acquisition time for each field was 0.5 s. The surface area for sperm head detection was set at 4 to 80 µm². The setup ensured that, in the majority of samples (11 out of 14), more than 1000 spermatozoa could be analyzed (mean of 1897 sperm).

Semen samples were assessed for the percentage of motile (total motility) and progressively motile spermatozoa (progressive motility). The following motility descriptors were recorded for each motile spermatozoon: straight line velocity (VSL), curved line velocity (VCL), average path velocity (VAP), linearity (LIN=VSL/VCL), straightness (STR=VSL/VAP), wobble (WOB=VAP/VCL), average amplitude of lateral head-displacement (ALH) and beat cross frequency (BCF). A spermatozoon was considered to be motile when it met one of the following three definitions: (1) average head orientation change (AOC) higher than 7° and BCF greater than 25 Hz; (2) DSL greater than 3.5 µm, VSL greater than 8 µm/s and DSL greater than 15 µm; or (3) VAP greater than 15 µm/s. Significance between wild-type and mutant spermatozoa was calculated using a paired Student's *t*-test (Table S1).

### Primers

Primers used were as follows: *cfap53* genotyping FW_*cfap53*, 5′-TGTAAGGAGAAGGAAGCAGGA; *cfap53* genotyping RV_*cfap53*, 3′-TCATCAATGCCCATCTGGTA; and *cfap53* genotyping in GFP-cfap53 transgenic background FW_*cfap53*, GGTGCTGGAGTTCACCAAAG; *cfap53* genotyping in GFP-cfap53 transgenic background RV_*cfap53* intron, 3′-ccgttcagacCTTTCCCTCT. Uppercase and lowercase letters indicate exons and introns, respectively.

## Supplementary Material

Supplementary information

Reviewer comments
