# Supplementary material for: The centriolar satellite protein Cfap53 facilitates formation of the zygotic microtubule organizing center in the zebrafish embryo
Source: Development. 2022 Aug 18;149(16):dev198762. doi: 10.1242/dev.198762 (PMC9481976; doi:10.1242/dev.198762)
Supplement: Supplementary information [file develop-149-198762-s1.pdf]

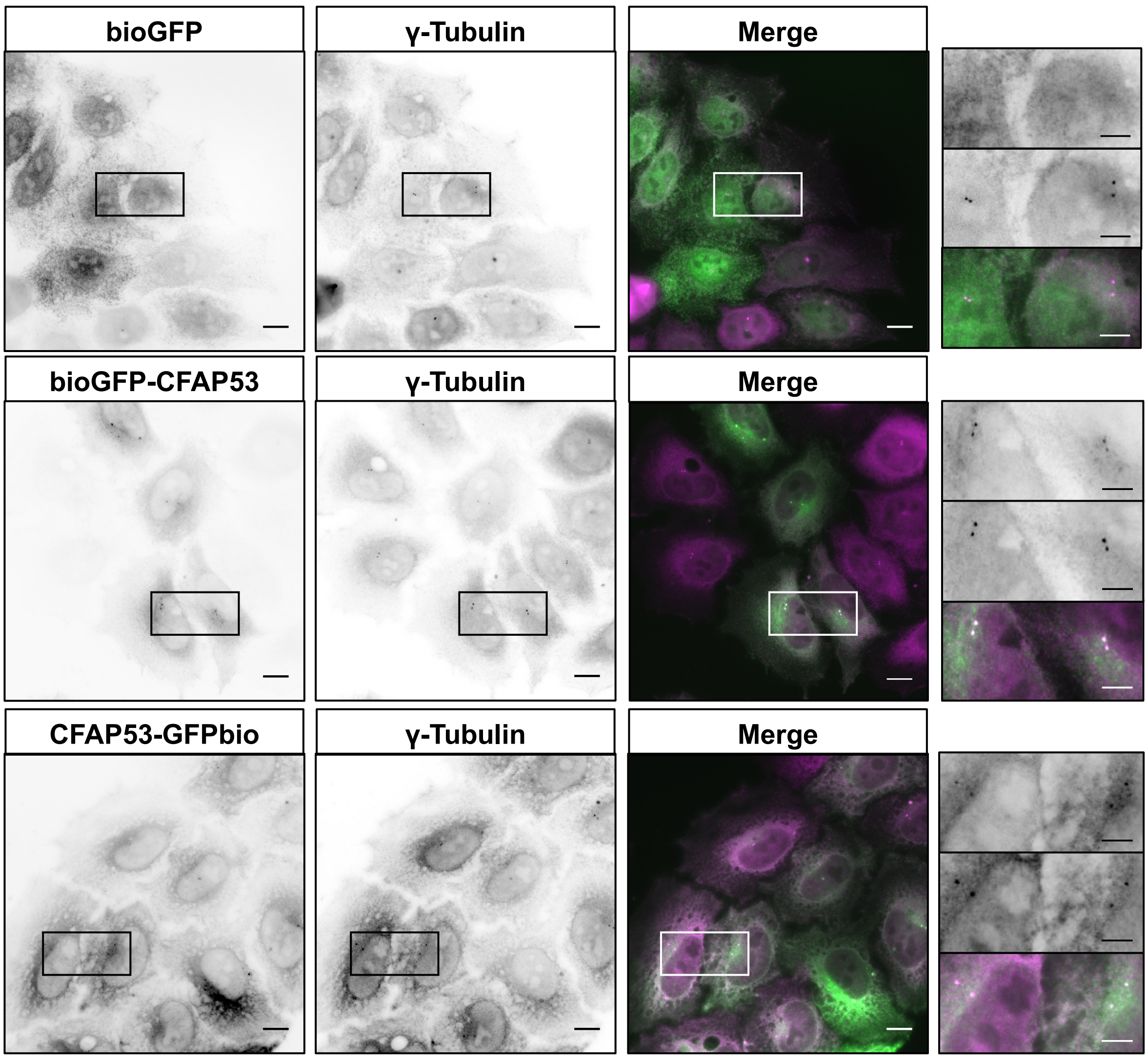

Fig. S1. GFP-CFAP53 localization in HeLa transfected cells. bioGFP-CFAP53 and CFAP53-GFPbio colocalize with γ-tubulin in transfected HeLa cells (n=25 for each construct).

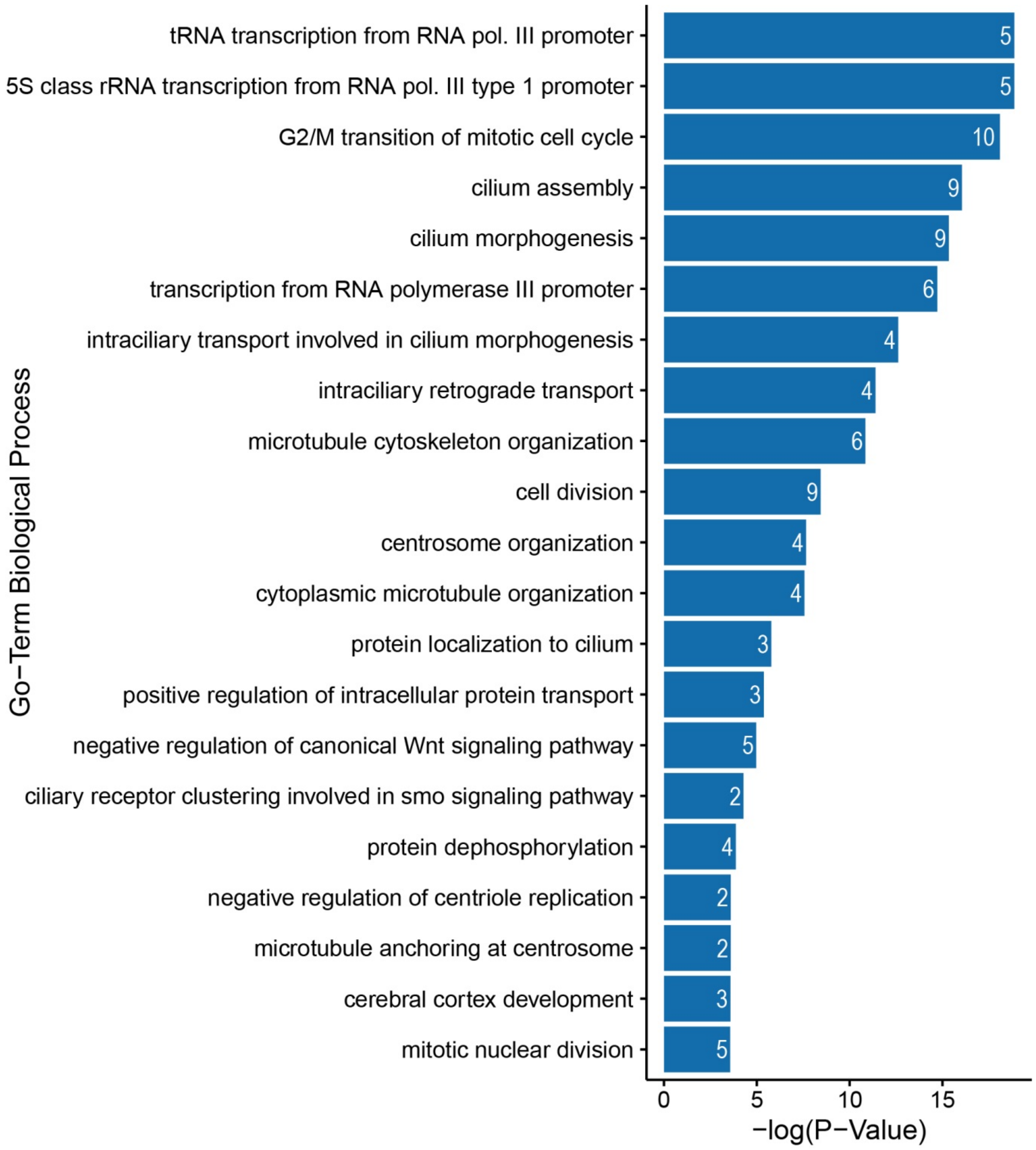

Fig. S2. Functional GO analysis reveals a high overrepresentation of centrosomal, microtubule and cell cycle related biological processes. High confident hits from the MS data set were selected and analyzed using DAVID thereby indicating highly enriched GO-Term biological processes in our MS data set(Huang et al., 2009; Sherman and Lempicki, 2009). Cutoff p-value < 0.03.

Development • Supplementary information

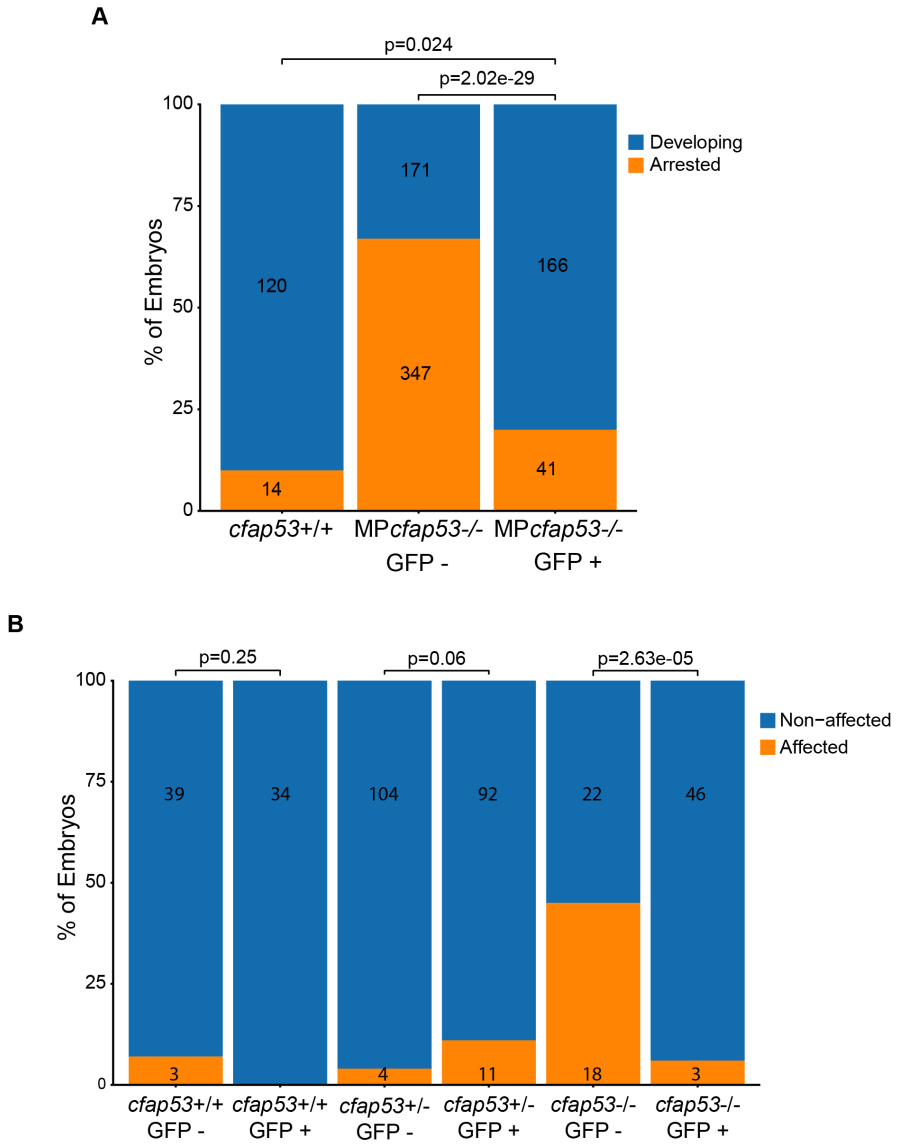

Fig. S3. GFP-Cfap53 is a fully functional fusion protein. (A) Barplot indicating the number of developing and arrested embryos of clutches derrived from wildtype, MP*cfap53* -/- or Tg(*ubi:GFP-Cfap53*)/MP*cfap53*-/- embryos analyzed at 45 mpf . Chi-squared test was used to test for significance (p-value <= 0.05).

(B) Barplot indicating the distribution of embryos from an incross of *cfap53* hetrozygous carriers with the Tg(*ubi:GFP-Cfap53*) that have affected laterality. Embryos with cardiac laterality defect (either right jogging or no jogging) were categorized as affected. Embryos with normal cardiac laterality (left jogging) were categorized as non-affected. Heart jogging and Cfap53-GFP expression was scored 26 hours after fertilization. As cardiac laterility is randomized in *cfap53*-/- embryos, only half of the mutant embryos are affected. P-value is generated from a test of independence (Fisher's Exact test). Numbers in bars indicate the number of embryos analysed from at least two independent matings.

Development • Supplementary information

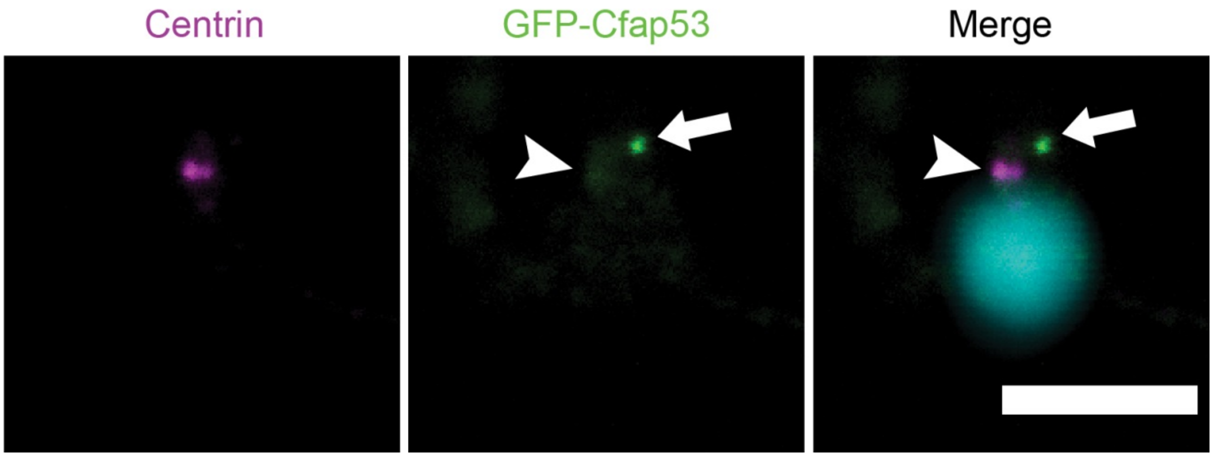

Fig. S4. GFP-Cfap53 localization in sperm. Centrin and GFP immunolabeling in fixed zebrafish sperm cells. In the merge DAPI is shown in cyan. Arrowhead indicates weak GFP-Cfap53 signal that colocalizes with Centrin. Arrow indicates strong signal of GFP-Cfap53 localizing in an uncharacterized structure in the sperm cell (n = 30 out of total 40 sperm cells analyzed in total). Scalebar indicates 5 microns.

Development • Supplementary information

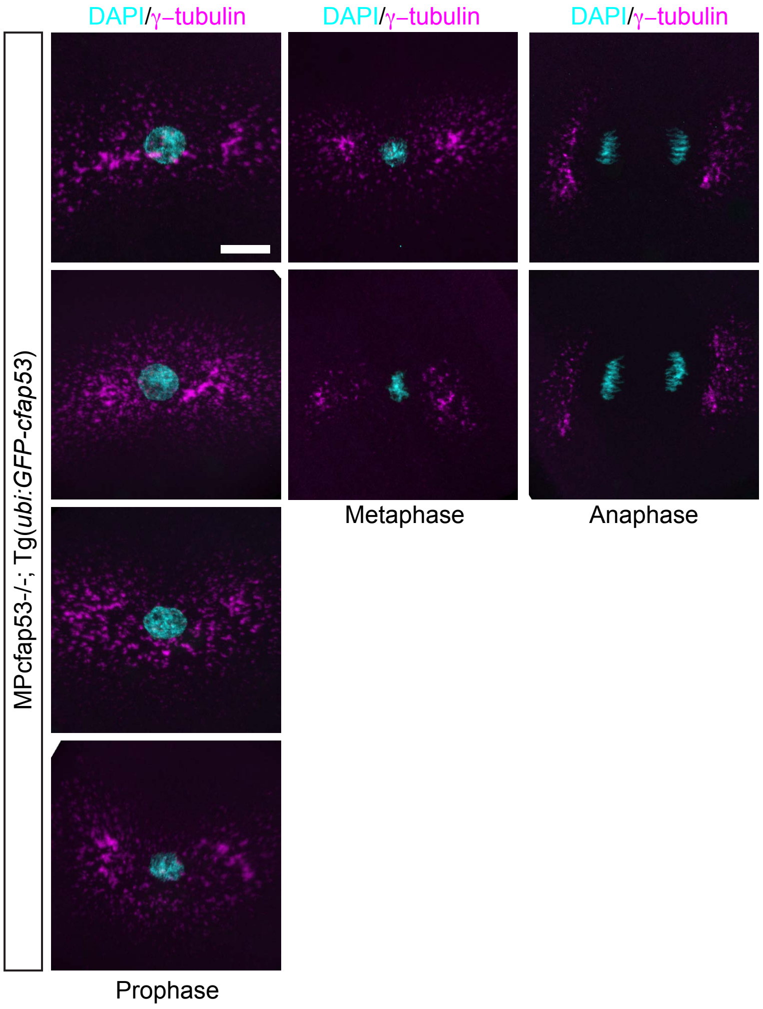

Fig. S5. Cfap53 is important for the first cell division.

Maximal projections of confocal stacks from fixed MP*cfap53-/-;Tg(ubi:GFP-cfap53)* embryos immunolabeled for γ-tubulin and DAPI at 45 mpf. γ-tubulin shows normal localization and chromosomes segregate normally during anaphase.

Development • Supplementary information

Table S1. Computer-assisted sperm analysis (CASA) of wild type and Cfap53 -/- sperm cells.

	Fish ID	Genotype	Total number of sperm cells	% Motile sperm cells
	2	mu	2606	76.5
	4	mu	1027	84.5
	6	mu	414	83.1
	8	mu	990	84.9
	10	mu	2432	78.9
	12	mu	2043	86.0
	14	mu	1450	85.5
**Mean**			1566	83.0
**SD**			819	4.0
	1	wt	1432	58.2
	3	wt	381	61.9
	5	wt	1052	87.1
	7	wt	2492	88.4
	9	wt	4981	83.5
	11	wt	1689	84.0
	13	wt	3565	87.8
**Mean**			2227	79
**SD**			1589	13
**T-test (p-value)**			0.347	0.437

Development • Supplementary information

Table S2: High confidence hits from GFP-CFAP53 and CFAP53-GFP AF-MS analysis.

Accession	gene name	Score BirA	Score GFP-Cfap53	Score Cfap53-GFP	FCA score	FCA>=7	FCB score	FCB>=3	SAINT probability	SAINT>=0.95
Q9Y5B8	NME7		9712.88	9373.19	195.86	TRUE	105	TRUE	1	TRUE
Q15154	PCM1		4291.57	6558.99	145.35	TRUE	22.58	TRUE	1	TRUE
O15078	CEP290		3566.09	4333.98	136.39	TRUE	82.03	TRUE	1	TRUE
Q12789	GTF3C1		1472.83	5518.23	112.92	TRUE	19.37	TRUE	1	TRUE
Q96M91	CFAP53	232.85	73292.73	52087.98	77.77	TRUE	186.39	TRUE	1	TRUE
Q14999	CUL7		3572.68	2817.23	75.71	TRUE	36.12	TRUE	1	TRUE
Q6P5Z2	PKN3		4494.97	1531.99	74.23	TRUE	39.55	TRUE	1	TRUE
Q8N4C6	NIN		2964.33	1122.01	66.11	TRUE	20.99	TRUE	1	TRUE
Q9Y2I6	NINL		1668.40	1813.86	62.64	TRUE	37.82	TRUE	1	TRUE
O94972	TRIM37		1930.66	915.02	51.14	TRUE	30.32	TRUE	1	TRUE
Q2M1P5	KIF7		1343.00	1718.19	49.87	TRUE	30.27	TRUE	1	TRUE
Q9UG01	IFT172		1476.88	1245.93	47.08	TRUE	20.13	TRUE	1	TRUE
A1XBS5	FAM92A1		2409.88	2791.52	44.58	TRUE	27.1	TRUE	1	TRUE
Q92621	NUP205	34.08	2351.65	3213.88	44.07	TRUE	7.71	TRUE	1	TRUE
Q9Y5Q9	GTF3C3		1317.28	2326.24	43	TRUE	8.85	TRUE	1	TRUE
Q9Y2K1	ZBTB1		1866.79	1530.88	40.22	TRUE	24.46	TRUE	1	TRUE
Q9Y5Q8	GTF3C5		666.78	1521.12	36.45	TRUE	9.07	TRUE	1	TRUE
Q9UKN8	GTF3C4		527.51	2123.97	33.64	TRUE	8.32	TRUE	1	TRUE
Q07864	POLE		428.18	1328.73	29.92	TRUE	10.67	TRUE	1	TRUE
Q96RY7	IFT140		888.15	658.20	29.03	TRUE	17.21	TRUE	1	TRUE
Q5TB80	CEP162		1131.49	472.55	28.72	TRUE	16.74	TRUE	1	TRUE
Q6ZU80	CEP128		622.24	506.35	28.4	TRUE	17.34	TRUE	1	TRUE
A6NKD9	CCDC85C		1361.54	787.12	28.1	TRUE	16.68	TRUE	1	TRUE
Q96C92	SDCCAG3		2080.95	1198.38	27.48	TRUE	13.04	TRUE	1	TRUE
O60318	MCM3AP		732.66	521.26	27.46	TRUE	13.31	TRUE	1	TRUE
Q9Y5P8	PPP2R3B		877.37	945.72	25.59	TRUE	15.72	TRUE	1	TRUE
Q9H6D7	HAUS4		1016.32	555.32	24.99	TRUE	6.91	TRUE	1	TRUE
Q9NVR7	TBCCD1		913.02	993.49	24.97	TRUE	15.34	TRUE	1	TRUE
Q13395	TARBP1		1281.56	480.86	24.37	TRUE	10.25	TRUE	1	TRUE
O95714	HERC2		198.09	748.55	23.08	TRUE	13.01	TRUE	1	TRUE
Q15058	KIF14		569.83	417.42	22.8	TRUE	5.48	TRUE	1	TRUE
Q9Y3M2	CBY1		1311.47	1305.70	22.79	TRUE	14.04	TRUE	1	TRUE
Q8TCG1	KIAA1524		932.99	516.93	20.93	TRUE	12.58	TRUE	1	TRUE
Q96JN8	NEURL4		613.28	817.72	20.92	TRUE	12.88	TRUE	1	TRUE
Q8WUA4	GTF3C2		182.82	1341.30	20.89	TRUE	7.15	TRUE	1	TRUE
O60232	SSSCA1		2125.04	293.58	20.64	TRUE	7.06	TRUE	1	TRUE
Q8NB25	FAM184A		728.91	408.52	20.63	TRUE	12.13	TRUE	1	TRUE
Q8TD10	MIPOL1		1092.86	565.77	20.31	TRUE	12.36	TRUE	1	TRUE

Q5SW79	CEP170		421.99	711.24	20.29	TRUE	3.67	TRUE	1	TRUE
Q9UL15	BAG5		1244.28	518.88	20.01	TRUE	9.23	TRUE	1	TRUE
Q53T94	TAF1B		625.19	1085.98	19.67	TRUE	12.09	TRUE	1	TRUE
Q9BV73	CEP250		568.26	385.68	19.37	TRUE	10.49	TRUE	1	TRUE
Q9UPN4	CEP131		494.31	455.41	19.36	TRUE	6.99	TRUE	1	TRUE
O14802	POLR3A		320.47	364.56	17.8	TRUE	6.13	TRUE	1	TRUE
Q86VW0	SESTD1		766.52	500.12	17.5	TRUE	10.87	TRUE	1	TRUE
P42695	NCAPD3		230.42	664.25	17.17	TRUE	7.04	TRUE	1	TRUE
Q7Z4Q2	HEATR3		603.73	494.12	16.56	TRUE	9.48	TRUE	1	TRUE
Q13136	PPFIA1		564.86	442.51	16.26	TRUE	7.98	TRUE	1	TRUE
Q7Z7A1	CNTRL		591.36	194.10	15.96	TRUE	8.23	TRUE	1	TRUE
Q96CS2	HAUS1		892.15	247.33	15.95	TRUE	8.72	TRUE	1	TRUE
Q12923	PTPN13		298.28	606.29	15.93	TRUE	9.6	TRUE	1	TRUE
Q15834	CCDC85B		523.45	259.31	15.33	TRUE	9.31	TRUE	1	TRUE
O94964	SOGA1		127.53	597.99	14.99	TRUE	6.44	TRUE	1	TRUE
Q92574	TSC1		829.10	229.59	14.71	TRUE	8.04	TRUE	1	TRUE
Q8N3Y1	FBXW8		608.23	810.83	14.69	TRUE	9.08	TRUE	1	TRUE
Q709F0	ACAD11		520.08	599.16	14.69	TRUE	9.08	TRUE	1	TRUE
Q96SN8	CDK5RAP2		297.33	346.72	14.39	TRUE	8.04	TRUE	1	TRUE
Q9NQC7	CYLD		512.00	183.38	13.77	TRUE	8.59	TRUE	1	TRUE
Q9UL42	PNMA2		579.18	357.88	13.77	TRUE	8.3	TRUE	1	TRUE
Q8NEZ3	WDR19		356.13	233.78	13.45	TRUE	8.42	TRUE	1	TRUE
P42345	MTOR		169.75	178.28	13.14	TRUE	4.12	TRUE	1	TRUE
Q86SQ0	PHLDB2		549.97	177.69	12.53	TRUE	7.06	TRUE	1	TRUE
Q9H1A4	ANAPC1		235.49	245.08	11.89	TRUE	7.5	TRUE	1	TRUE
Q8IWT3	CUL9		650.78	583.43	11.89	TRUE	4.92	TRUE	1	TRUE
P23258	TUBG1		509.59	288.73	11.59	TRUE	4.89	TRUE	1	TRUE
Q7Z494	NPHP3		594.04	26.64	11.29	TRUE	4.68	TRUE	1	TRUE
Q8TED0	UTP15		131.05	455.84	10.95	TRUE	5.61	TRUE	1	TRUE
Q7Z4L5	TTC21B		254.32	147.79	10.66	TRUE	6.46	TRUE	1	TRUE
P07199	CENPB		519.75	331.42	10.35	TRUE	3.32	TRUE	1	TRUE
Q06190	PPP2R3A		290.99	276.44	10.34	TRUE	6.59	TRUE	1	TRUE
Q9UPU5	USP24		376.51	133.43	10.03	TRUE	4.36	TRUE	1	TRUE
Q96GE4	CEP95		316.56	288.25	10.02	TRUE	6.38	TRUE	1	TRUE
Q9HD67	MYO10		201.65	201.65	9.71	TRUE	6.21	TRUE	1	TRUE
Q9C099	LRRCC1		215.78	89.83	9.42	TRUE	4.89	TRUE	1	TRUE
Q9HBG6	IFT122		264.23	136.12	8.47	TRUE	5.36	TRUE	1	TRUE
Q9BWT7	CARD10		153.06	119.83	8.16	TRUE	5.26	TRUE	1	TRUE
Q15029	EFTUD2		181.21	282.05	8.15	TRUE	4.23	TRUE	1	TRUE
Q8WV44	TRIM41		64.95	368.58	8.15	TRUE	3.31	TRUE	1	TRUE
A1A4S6	ARHGAP10		382.43	32.38	7.86	TRUE	3.84	TRUE	1	TRUE
P14373	TRIM27		283.78	223.57	7.85	TRUE	3.36	TRUE	1	TRUE

Development • Supplementary information

Q9Y4C2	TCAF1		240.29	169.07	7.54	TRUE	4.91	TRUE	1	TRUE
Q5JTC6	AMER1		283.72	214.16	7.54	TRUE	3.67	TRUE	1	TRUE
Q8NCN5	PDPR		308.17	126.01	7.23	TRUE	4.73	TRUE	1	TRUE
Q06481	APLP2		275.02	113.53	7.23	TRUE	4.67	TRUE	1	TRUE
O00743	PPP6C		219.78	106.47	7.23	TRUE	4.59	TRUE	1	TRUE
Q9GZT3	SLIRP		270.85	142.26	7.23	TRUE	4.35	TRUE	1	TRUE
Q96RT7	TUBGCP6		237.52	201.74	7.23	TRUE	3.97	TRUE	1	TRUE
P51530	DNA2		153.36	178.44	7.22	TRUE	4.71	TRUE	1	TRUE

Development • Supplementary information

Table S3. Overlap of identified proteins in this study compared to two centriolar satellite proteomic studies.

	Overlap with this study (%)
**Quarantotti et al., 2019**	28
**Gheigratmand et al., 2019**	38

**Overlapping proteins with this study**	
**Quarantotti et al. 2019**	**Gheigratmand et al., 2019**
PCM1	NME7
CEP290	PCM1
NIN	CEP290
TRIM37	NIN
KIF7	NINL
CEP162	TRIM37
CEP128	KIF7
PPP2R3B	CEP128
HAUS4	SDCCAG3
HERC2	MCM3AP
KIF14	HAUS4
CBY1	HERC2
FAM184A	KIF14
CEP170	KIAA1524
CEP250	NEURL4
CEP131	FAM184A
CNTRL	MIPOL1
HAUS1	BAG5
ACAD11	CEP250
CDK5RAP2	CEP131
CYLD	PPFIA1
TUBG1	HAUS1
CEP95	PTPN13
LRRCC1	SOGA1
TRIM41	TSC1
	CDK5RAP2
	CYLD
	PHLDB2
	PPP2R3A
	CEP95
	LRRCC1
	TRIM41
	TRIM27

Development • Supplementary information
