# Supplementary material for: The centriolar satellite protein Cfap53 facilitates formation of the zygotic microtubule organizing center in the zebrafish embryo
Source: Development. 2022 Aug 18;149(16):dev198762. doi: 10.1242/dev.198762 (PMC9481976; doi:10.1242/dev.198762)
Supplement: Reviewer comments [file develop-149-198762_review_history.pdf]

The centriolar satellite protein Cfap53 facilitates formation of the zygotic microtubule organizing center in the zebrafish embryo

Sven Willekers, Federico Tessadori, Babet van der Vaart, Heiko H. Henning, Riccardo Stucchi, Maarten Altelaar, Bernard A. J. Roelen, Anna Akhmanova and Jeroen Bakkers
DOI: 10.1242/dev.198762

Editor: Steve Wilson

Review timeline

Original submission:	18 November 2020
Editorial decision:	1 September 2021
First revision received:	18 January 2022
Editorial decision:	3 March 2022
Second revision received:	25 May 2022
Editorial decision:	4 July 2022
Third revision received:	18 July 2022
Accepted:	20 July 2022

Original submission

First decision letter

MS ID#: DEVELOP/2020/198762

MS TITLE: The centriolar satellite protein Cfap53/Ccdc11 facilitates the formation of the first zygotic microtubule organizing center in the zebrafish embryo

AUTHORS: Sven Willekers, Federico Tessadori, Babet van der Vaart, Heiko H.W. Henning, Riccardo Stucchi, Maarten Altelaar, Bernard A.J. Roelen, Anna Akhmanova, and Jeroen Bakkers

Dear Jeroen,

Best wishes for the new year. I have now received all the referees' reports on the above manuscript, and have reached a decision. The referees' comments are appended below, or you can access them online: please go to BenchPress and click on the 'Manuscripts with Decisions' queue in the Author Area.

As you will see, the referees express considerable interest in your work, but have some significant criticisms and suggestions for improvements to your manuscript. If you are able to revise the manuscript along the lines suggested, I will be happy receive a revised version of the manuscript. Please also note that Development will normally permit only one round of major revision.

We are aware that you may be experiencing disruption to the normal running of your lab that make experimental revisions challenging. If it would be helpful, we encourage you to contact us to discuss your revision in greater detail. Please send us a point-by-point response indicating where you are able to address concerns raised (either experimentally or by changes to the text) and where you will not be able to do so within the normal timeframe of a revision. We will then provide further guidance. Please also note that we are happy to extend revision timeframes as necessary.

Please attend to all of the reviewers' comments and ensure that you clearly highlight all changes made in the revised manuscript. Please avoid using 'Tracked changes' in Word files as these are lost

© 2022. Published by The Company of Biologists under the terms of the Creative Commons Attribution License (https://creativecommons.org/licenses/by/4.0/).

in PDF conversion. I should be grateful if you would also provide a point-by-point response detailing how you have dealt with the points raised by the reviewers in the 'Response to Reviewers' box. If you do not agree with any of their criticisms or suggestions please explain clearly why this is so.

With best wishes,

Steve

Stephen Wilson
Handling Editor
Development

Reviewer 1

Advance summary and potential significance to field

Willekers et al describe an interesting maternal-paternal phenotype in initiating MTOC organization in the zebrafish embryo. Their data presentation is clear and concise and the novelty of this variably penetrant phenotype and its genetics is noteworthy.

Summary of advance: The authors identify a requirement for maternal and paternal cfap53 in formation of the MTOC in the early zebrafish embryo. This identifies unique aspects of the first division of the zygote and support for essential paternal contributions to this process.

Comments for the author

I'm curious about if and when the cell cycle is actually arrested in these mutants. The authors describe the first indication of localization of GFP cfap53 during early metaphase. They also describe incorporation of EdU in arrested embryos indicating that embryos initiate S phase. Is there an MTOC checkpoint prior to condensation of chromatin in M phase? Anti phospo histone staining would help to narrow down the point of arrest. Alternatively, the zygote nucleus may be undergoing endoreduplication. Adding EdU later would indicate if the embryos are truly arrested in the cell cycle or if they just fail to initiate karyokinesis and cytokinesis but continue to cycle. Some images of mutant nuclei appear to be condensed while others seem fragmented, indicating a mitotic catastrophe.
Some sections require clarification:
This sentence isn't clear to me: "Streptavidin-based pull downs of both fusion proteins co-expressed with biotin ligase BirA or biotin ligase BirA expression alone as a negative control followed by MS resulted in the retrieval of in total 1148 overlapping proteins.". Which samples are overlapping? The N and C fusions or the HeLa and HEK293 cells?
In the sentence, "From this extensive analysis 88 proteins were considered putative binding partners of Cfap53 (Table S2)", it is not clear how these 88 proteins were selected.
In the discussion much is made of the novelty of the initiation of the MTOC in the early embryo because of the large size of the cell. The yolk syncytial nuclei also have large diffuse MTOCs and long arrays of microtubules but no defect is described in these. If there is no defect in the YSN this indicates that the key step is initiation of the MTOC from gamete components rather than the size of the cell exclusively. It may be worth mentioning this for some perspective in the discussion.
There are a few copy editing points to consider:
"(1.000x in case of the zebrafish)" in UK and USA English the number 1,000 either uses a comma or no unit separator In the legend of figure 3 "Proteins shows are proteins with" I think should read "proteins shown are those with"

Reviewer 2

Advance summary and potential significance to field

The authors present work that describes a novel role for cfap53 in establishing a functional MTOC during the first cell division in developing zebrafish embryos. They have discovered that the loss of maternal and paternal cfap53 leads to embryos that fail to undergo cell division during the first

© 2022. Published by The Company of Biologists under the terms of the Creative Commons Attribution License (https://creativecommons.org/licenses/by/4.0/).

mitosis in zebrafish embryogenesis, an aberrant phenotype that the authors link to an irregular MTOC in maternal-paternal cfap53 loss-of-function mutants. The first mitotic division is a critical stage in embryonic development and understanding how this process is faithfully executed is of broad interest to the developmental biology community and of particular interest to researchers that study early embryonic development. This makes this discovery of a new role for cfap53 relevant and of interest to a broad audience of Development readers.

Although the work presented would make a significant contribution to understanding developmental mechanisms early in embryogenesis, there are some major and minor concerns that need to be addressed. Overall, the research presented was clear and easy to follow (except for the instances mentioned below). Satisfactory efforts to resolve the concerns mentioned below would make the manuscript well suited for publication in Development.

Comments for the author

Major concerns:
1) In figure 1C, it wasn't clear if embryos for a single pair of fish were used to generate the data for each bar of the graph or if the data from each bar are from embryos of different pairs of fish. For example, is the data from the cross between the cfap53-/- female and the WT male from the same pair of fish or is it from different fish paired together with the same genotypes? Given the large number of embryos counted, it seems as though different pairs of crosses were used per category on the x-axis, but I did not find this information within the text. In terms of confidence in reproducibility, it is important to show that the data is a quantification of at least 3 different pairings per category.
2) In figure 1E, is there a subset of the MPcfap53-/- embryos, presumably the developing MPcapf53-/- embryos, that look like WT during 30 mpf and 40 mpf? Based on Figure 1C, 30% or more are expected to look like WT. It is important to report on this and include these images to show, in agreement with Figure 1A, that the developing MPcfap-/- embryos indeed look like WT in both a macroscopic and microscopic scale. Again please report on the number of different MP fish pairs tested, at least two are needed.
3) How was the EdU experiment in Figure 2E performed? The authors cite Dekens et al. 2003 in the results section but do not include their own methodology in their own section on materials and methods. Also, it is important to include a control using activated squeezed eggs that are unfertilized to confirm that EdU would not incorporate in the DNA of unfertilized embryos. While I understand that the thymidine analog is unlikely to incorporate in the DNA of the unfertilized egg, it would be best to show this comparison with the MPcfap53-/- embryos, especially since it is a feasible experiment to perform.
4) The images for the developing MPcfap53-/- embryos should also be included with Figure 5D-I. Excluding the developing MPcfap53-/- from the staining in Figure 5D-I risks missing phenotypes that might differ from WT or phenotypes that might be similar to the arrested MPcfap53-/- embryos.
5) There is no gamma-tubulin staining in 5A-C but the authors state there is in the results section. This should be corrected in either the text or the image data. Also, PCM1 co-staining with Cfap53 should be performed since the authors are suggesting there is an interaction occurring between these proteins based on their MS data.
6) In some panels in Fig 5, the MTOCs are not positioned in line with the metaphase chromosomes, as normally is the case (e.g. D, E). This may be due to a mounting issue, if the 'bridging' method used to mount the cover slip applies pressure and squishes a bit the cells, causing displacement of the MTOC from the metaphase plate. This can also cause micronuclei to disperse as well and could in part cause the phenotype in G.
7) Were the embryos that were used for Figure 6 selected at random? There is an n = 6 shown for both the developing and arrested embryos, which does not agree with the ratios of developing to arrest embryos seen in other figures. Can the authors explain this possible discrepancy?
8) The authors state that the GFP-Cfap53 transgene rescues left-right abnormalities and direct the reader to Figure S3, yet these data are not present in Figure S3. Please correct. The visual data would add to the quality of the rescue.
9) At least one analysis of gamma-tubulin, PCM1, or Centrin (similar to experiments in Figure 5D-I) should be performed on the MPcfap53-/- embryos that are rescued by the transgenic GFP-Cfap53 to show the extent of rescue.
10) The text in the results section (third sentence below "Cfap53 interactome" section title) says streptavidin-pulldown and cell culture work was done with both zebrafish Cfap53 and human

© 2022. Published by The Company of Biologists under the terms of the Creative Commons Attribution License (https://creativecommons.org/licenses/by/4.0/).

CFAP53. I did not find evidence of human CFAP53 being used in either the Material and Methods section or the figures. This should be clarified or corrected.
11) Regarding analysis in figure 7, it is stated that the treatments do not effect g-Tubulin localization to the MTOC, but no secondary marker of the MTOC is examined. Either examine g-Tubulin with another MTOC marker or modify the conclusions.

Minor:
1) Authors state that “Cfap53 is required to initiate the first cell division,” but saying required is a strong statement since some embryos do divide. Please reword. For example, the authors use “facilitates” in the title and in other areas in the results section.
2) The GFP-Cfap53 that is stated to be co-localizing with Centrin in Figure S4 (arrowhead) looks similar to the background GFP staining to the left of the arrowhead. Are there clearer images to make this point more convincing?
3) What was used as an indicator for selecting arrested embryos at 30 mpf in Figure 6? Was there a clear distinction between the developing and arrested embryos at this time-point other than the staining results? Perhaps including an image of the 30 mpf embryos in Figure 1A would be helpful.
4) Was immunolabeling for Cfap53 also performed for the nocadazole treatment experiments shown in Figure 7? If so, it would be valuable to include this data to show whether Cfap53 localization is also affected similar to gamma-tubulin (since the authors show that both proteins co-localize in Figure 4). Dispersion of Cfap53 due to destabilizing microtubules would agree with experiments performed in Silva et al. 2016. Further demonstrating the behavior and similarity of Cfap53 between different systems.
5) Figure 2, indicate the number of males or MPcap53 male/female pairs tested? At least 2 different males and MP pairs should be tested, and 3 is best. Repeat if not two biological replicates and indicate the number of embryos tested for each.
6) Figure 2A, B, the males should be just cfap53-/- rather than MPcfap53, correct?
7) In Fig 2E, why is the nuclear morphology so different to Fig 1E. Please comment on this in the text.
8) Figure 4, it is a bit confusing to have a nucleus or nuclei depicted in the left schematics, when nuclei are not actually present at metaphase, anaphase stages. Please modify.
9) Page 6 “As unfertilized oocytes arrest in a similar manner,”, once an oocyte passes through the oviduct, it is an egg. Change unfertilized oocytes to eggs. Same issue on p9, first paragraph, oocyte should be egg. And again on p15.
10) On page 9, provide a reference for this statement during the cleavage stage: “Failure in MTOC formation will result in a disorganized microtubule network and cell cycle arrest, since proper bipolar spindles cannot be formed.”
11) Provide N values for Fig S1, S3 S4 for adult tested (at least two needed), cells or sperm examined.
12) Page 14, 1.000x change to 1,000x.

<u>Reviewer 3</u>

Advance summary and potential significance to field

The manuscript by Willekers et al documents the characterization of a maternal and paternal functions for the satellite protein Cfap52/Cdc11 in the zebrafish embryo. This gene has been previously shown to have effects zygotically in embryo laterality, and the authors now study an effect observable in the offspring of homozygous mutant embryos, revealing previously unknown functions in the gametes. The work also includes proteomic analysis to establish networks of protein interactions for this factor. The parental function of this gene is of interest and in addition the authors suggest a function for the gene specifically in the first mitotic cycle, which would be a novel type of function (see below, additionally including caveats for this interpretation).

Comments for the author

There are some concerns with the manuscript
Controls are not well documented. One set of controls involves the known fact that zebrafish fertilization rates during natural pair matings are known to vary widely from 0 to 100%. Thus, it is important to have clear and stringent protocols to address this potential variability, yet these are

© 2022. Published by The Company of Biologists under the terms of the Creative Commons Attribution License (https://creativecommons.org/licenses/by/4.0/).

not well documented. It is unclear for example how many fish or crosses are used for the various experiments. Additionally, as the authors point out, embryos that lack a division in the first cell cycle are not readily distinguishable from unfertilized embryos. The primary strategy in this work to address this is to use a time course of fixed embryos, where the authors observe a normal fraction of pronuclei and pronuclear fusion in early stages – however there is not a direct correlation between the time course and the live phenotype (e.g. the same clutch(es) being used for both experiments). In the absence of a direct effort to address this, it is difficult to know whether the observed data is simply a consequence of clutch variability, rather than the authors' interpretation.
Another set of controls that are missing from the paper are generally, wild-type embryos (the authors rely on the unaffected mutant embryos for this) as well as unfertilized controls.
Related to the last point, the authors should make a stronger case for the mutant arrested embryos not being unfertilized embryos. An example of the superficial way in which this is addressed (in addition to the time course of fixed embryos with technical caveats as stated above) is the fact that the authors use a DNA incorporation argument to support the idea that the mutants are not unfertilized embryos, since the authors see incorporation of EdU in mutant embryos but a previous study (Dekens et al 2002) had shown that unfertilized embryos do not. However, the Dekens et al used a different compound (BrdU) and it is unclear how comparable the methods used are (in particular the EdU protocol in this manuscript is missing in the Methods section). Even if the protocols were comparable, given the importance of the issue (unfertilized vs arrested) to the logical core of this study, the manuscript should include parallel studies on unfertilized embryos as controls/comparison.
The authors propose a model in which Cfap53 is required parentally only for the first mitotic division. This is a very unexpected discrete functional period for two reasons. First, centrosome and aster formation in the monoester involved pronuclear fusion are generally thought to be similar to those of the initial mitotic spindles, with the primary difference being the number of centriole pairs leading to a monoester vs bipolar conformation. If the first mitotic spindle but not the monoaster are affected, this would be to my knowledge the first study that shows functions specific for these two types of structures. The idea that the bipolar spindle is specifically affected is supported by the observation of Cfap53 appears localized during mitoses but not in the early embryo immediately after fertilization (however, this idea contrasts with the observation of Cfap53 in sperm). However, visualization of Cfap53 relies on a GFP construct, and there may be a lag in translation of maternal GFP protein after fertilization leading to the absence of labeling in the monoaster as a potential artefact. These potential caveats and complexities are not clearly addressed in the manuscript.
A second reason a discrete function only in the first mitosis is unexpected is that generally studies have shown that the mitoses in the early embryo are similar and part of a protracted egg-to-embryo transition that lasts into the cleavage stages. This proposed first-mitosis-only discrete function also conflicts with the observation of Cfap53 in the centrosomes in later stages of the cell cycle. Given this localization, a reasonable expectation would be that Cfap53 functions through the early cleavage early mitoses, not just the first one. Given that the arrest at the first cell cycle precludes observing the subsequent cycles, the authors seem to rely on several arguments to support a first-mitosis-only function. The first argument seems to be that the fraction of embryos that does not exhibit the arrest continue to divide normally – however, this can be explained by incomplete penetrance (see below) rather than temporal selectivity. A second argument is the differential effect of nocodazole treatment during the early period (0-45 min), which disrupts gamma-tubulin localization, compared to a later period (the third mitosis only), which does not. However, these treatments are of different length. Additionally, the authors do not state a dechorionation step prior to drug treatment (Methods) and the chorion is known to be a partial barrier to nocodazole. Given that the chorion's structure and impermeability develops during egg activation, it could well be that the observed differential effect is due to the differential permeability of the chorion at early to later stages, in addition to the difference in time length of treatment.
Additionally, the observed fraction of embryos that are not affected by Cfap53 loss of function suggests that the function is not fully essential and that the embryos are in a functional threshold range leading to incomplete penetrance of the phenotype. Given this incomplete penetrance and near-threshold activity, one would also expect that the first cell division, which involves the largest cells and internal cellular structures, would be the most sensitive to functional reduction, leading to phenotypes that may be observable only in the first mitosis and not in subsequent mitosis. This is thought to be the case for example with the janus mutation, which preferentially affects the

© 2022. Published by The Company of Biologists under the terms of the Creative Commons Attribution License (https://creativecommons.org/licenses/by/4.0/).

first cell division, likely because of sub-optimal cell-cell adhesion leading to a separation of the first (largest and needing greater cohesion) blastomeres.
Regarding the paternal effect, there is no control for the males' mating behavior. Zebrafish fertilization occurs in a tight window where sperm ejection needs to be in close proximity of the eggs, with sperm entry needed before the post activation sperm blocks occurs. Thus any changes in behavior could increase the fraction of unfertilized eggs, which given uncertainties as stated above could be confused with arrested embryos. Given that the males have laterality defects, it is possible that they could have defects in mating behavior. This could be controlled using in vitro fertilization, though in this case sperm function and concentration would have to be comparable.
In the Discussion: "Why these structures that function as MTOCs are so large is not clear, but might have to do with the large size of the embryo (see also below)". There is extensive literature documenting that given the limited maternal pools for products needed for cell division, cellular structures are large at first and become smaller gradually as they adjust to smaller cell sizes (cellular scaling, see for example literature by M. Wühr).

First revision

Author response to reviewers' comments

We like to thank all reviewers for their efforts and their constructive feedback on our manuscript. We have addressed all questions and points raised and our response is added in blue text. In the manuscript file the changes to the text are highlighted in blue text. Unfortunately, these revisions took much longer than anticipated, which is mainly due to covid restrictions and related issues due to the covid pandemic. We want to thank the reviewers for their patience.

Reviewer 1
Advance Summary and Potential Significance to Field:
Willekers et al describe an interesting maternal-paternal phenotype in initiating MTOC organization in the zebrafish embryo. Their data presentation is clear and concise and the novelty of this variably penetrant phenotype and its genetics is noteworthy.

Summary of advance: The authors identify a requirement for maternal and paternal cfap53 in formation of the MTOC in the early zebrafish embryo. This identifies unique aspects of the first division of the zygote and support for essential paternal contributions to this process.

Reviewer 1 Comments for the Author:
I'm curious about if and when the cell cycle is actually arrested in these mutants. The authors describe the first indication of localization of GFP cfap53 during early metaphase. They also describe incorporation of EdU in arrested embryos indicating that embryos initiate S phase. Is there an MTOC checkpoint prior to condensation of chromatin in M phase? Anti phospo histone staining would help to narrow down the point of arrest. Alternatively, the zygote nucleus may be undergoing endoreduplication. Adding EdU later would indicate if the embryos are truly arrested in the cell cycle or if they just fail to initiate karyokinesis and cytokinesis but continue to cycle. Some images of mutant nuclei appear to be condensed while others seem fragmented, indicating a mitotic catastrophe.

Response: The reviewer addresses an important point. (S)he suggests to perform EdU labeling experiments starting at 45 min to test whether arrested mutant embryos continue to cycle and undergo endoreduplication. We performed the suggested EdU labeling experiment and the results are summarized in Table 2b. We found that in wild type embryos 77% (n=40/52) of the normally developing embryos incorporated EdU. The fact that 23% of wt developing embryos fail to incorporate EdU is likely due to a technical issue with EdU penetrating the embryo. In contrast, we never observed EdU incorporation in unfertilized eggs (n=0/26). Importantly, 50% (n=22/44) of the arrested MPcfap53-/- embryos continue to incorporate EdU even after 45 mpf and in the absence of cytokinesis. The experiment was performed with 4 independent lays from different pairs resulting in comparable results from the different lays. These results indicate that fertilized (EdU+) but

© 2022. Published by The Company of Biologists under the terms of the Creative Commons Attribution License (https://creativecommons.org/licenses/by/4.0/).

arrested MPcfap53 mutant embryos fail to initiate karyokinesis and cytokinesis, but continue to replicate their DNA.

We have incorporated these data in the results section on page 7:

Some sections require clarification:
This sentence isn't clear to me: "Streptavidin-based pull downs of both fusion proteins co-expressed with biotin ligase BirA or biotin ligase BirA expression alone as a negative control followed by MS resulted in the retrieval of in total 1148 overlapping proteins.". Which samples are overlapping? The N and C fusions or the HeLa and HEK293 cells?

Response: The overlapping samples refers to the samples from the N- and C-terminal fusion proteins. We removed references to the HeLa cells as this was confusion and modified the text on page 7 to make it more clear:
'*Streptavidin-based pull downs followed by MS resulted in the retrieval of in total 1148 proteins that were pulled down by both the bioGFP-CFAP53 and the CFAP53-bioGFP.*'

In the sentence, "From this extensive analysis 88 proteins were considered putative binding partners of Cfap53 (Table S2)", it is not clear how these 88 proteins were selected.

Response: This was described in the Materials and Methods section: To retrieve significant putative binding partners of CFAP53 additional analyses on the raw MS data were performed using Contaminant Repository for Affinity Purification (the CRAPome) implementing fold change scoring (FC) scoring and probalistic scoring using SAINT. The FC is calculated based on the ratio of average normalized spectral counts in bait purifications to negative controls. SAINT uses statistical modeling which provides a probability of true interaction (Choi et al., 2011; Mellacheruvu et al., 2013). The FC scoring is subdivided into a FCA (primary score based on BirA negative control pull down) and FCB (secondary score based on existing GFP negative control). The FCA and FCB cut-off were set to seven and three. The SAINT probability cut-off was set to 0.95. These settings resulted in the list of 88 proteins provided in Table S2.

In the discussion much is made of the novelty of the initiation of the MTOC in the early embryo because of the large size of the cell. The yolk syncytial nuclei also have large diffuse MTOCs and long arrays of microtubules but no defect is described in these. If there is no defect in the YSN this indicates that the key step is initiation of the MTOC from gamete components rather than the size of the cell exclusively. It may be worth mentioning this for some perspective in the discussion.

Response: While we found several papers describing the long arrays of microtubules that are present in the YSL (e.g. PMID: 7956824; PMID: 8187646), we could not find papers that characterize the MTOC in the YSL using antibodies against gamma-tubulin or Centrin. In our own immunostainings with gamma-tubulin or Centrin of blastula stage embryos we did not observe large variations in MTOCs sizes and did not observe MTOCs that were as large as the MTOC at the first mitosis (compare Fig. 4C and F). If the reviewer feels that we missed some important literature please clarify which studies should be included.

There are a few copy editing points to consider:"(1.000x in case of the zebrafish)" in UK and USA English the number 1,000 either uses a comma or no unit separator

Response: We thank the reviewer for pointing this out and we have now changed this in 1,000x

In the legend of figure 3 "Proteins shows are proteins with" I think should read "proteins shown are those with"

Response: This mistake is now corrected.

Reviewer 2
Advance Summary and Potential Significance to Field:
The authors present work that describes a novel role for cfap53 in establishing a functional MTOC during the first cell division in developing zebrafish embryos. They have discovered that the loss of maternal and paternal cfap53 leads to embryos that fail to undergo cell division during the first

© 2022. Published by The Company of Biologists under the terms of the Creative Commons Attribution License (https://creativecommons.org/licenses/by/4.0/).

mitosis in zebrafish embryogenesis, an aberrant phenotype that the authors link to an irregular MTOC in maternal-paternal cfap53 loss-of-function mutants. The first mitotic division is a critical stage in embryonic development and understanding how this process is faithfully executed is of broad interest to the developmental biology community and of particular interest to researchers that study early embryonic development. This makes this discovery of a new role for cfap53 relevant and of interest to a broad audience of Development readers.

Although the work presented would make a significant contribution to understanding developmental mechanisms early in embryogenesis, there are some major and minor concerns that need to be addressed. Overall, the research presented was clear and easy to follow (except for the instances mentioned below). Satisfactory efforts to resolve the concerns mentioned below would make the manuscript well suited for publication in Development.

Reviewer 2 Comments for the Author:
Major concerns:
1) In figure 1C, it wasn't clear if embryos for a single pair of fish were used to generate the data for each bar of the graph or if the data from each bar are from embryos of different pairs of fish. For example, is the data from the cross between the cfap53-/- female and the WT male from the same pair of fish or is it from different fish paired together with the same genotypes? Given the large number of embryos counted, it seems as though different pairs of crosses were used per category on the x-axis, but I did not find this information within the text. In terms of confidence in reproducibility, it is important to show that the data is a quantification of at least 3 different pairings per category.

Response: For the data shown in Fig.1C we used multiple pairings of a single male and female fish. The reason for taking a single male and female was that we observed variation in the penetrance of the phenotype when using different males and females and wanted to minimize this when looking at the difference between the paternal and maternal contribution to the phenotype. We have made this now clear in the figure legend. We agree with the reviewer that in terms of reproducibility it is important to have quantification of the phenotype from different pairs of fish. We therefore included an additional table (Table1) in which we provide a quantification of the phenotype using at least 3 different pairs. In addition, the congression and EdU labeling experiments were performed on embryos from mating at least 3 different pairs (indicated in figure legends).

2) In figure 1E, is there a subset of the MPcfap53-/- embryos, presumably the developing MPcapf53-/- embryos, that look like WT during 30 mpf and 40 mpf? Based on Figure 1C, 30% or more are expected to look like WT. It is important to report on this and include these images to show, in agreement with Figure 1A, that the developing MPcfap-/- embryos indeed look like WT in both a macroscopic and microscopic scale. Again please report on the number of different MP fish pairs tested, at least two are needed.

Response: The time course experiment shown in Figure 1D,E was performed on lays of wild type and cfap53 mutant fish. The images shown in Figure 1D were taken from the wild type embryos while images shown in Figure 1E were taken from the embryos derived from the cfap53 mutant parents. Up to 20 mpf we did not observe any differences in the clutch of MPcfap53 embryos and all looked comparable to their wild type controls. From 30 mpf we started to see differences in the MPcfap53 clutch with some embryos normally proceeding through karyogenesis and cytokinesis while other embryos failed to do so. In the original figure we only showed the pictures of the embryos with failed karyogenesis/cytokinesis at 30 mpf and 45 mpf. We now have included pictures of MPcfap53 embryos that showed normal karyogenesis/cytokinesis.
Results were obtained from 2 *cfap53-/-* pairs, which is included now in the legend

3) How was the EdU experiment in Figure 2E performed? The authors cite Dekens et al. 2003 in the results section but do not include their own methodology in their own section on materials and methods. Also, it is important to include a control using activated squeezed eggs that are unfertilized to confirm that EdU would not incorporate in the DNA of unfertilized embryos. While I understand that the thymidine analog is unlikely to incorporate in the DNA of the unfertilized egg, it would be best to show this comparison with the MPcfap53-/- embryos, especially since it is a feasible experiment to perform.

© 2022. Published by The Company of Biologists under the terms of the Creative Commons Attribution License (https://creativecommons.org/licenses/by/4.0/).

Response: We apologize for omitting a detailed description of the EdU labeling experiment in the original manuscript. We now included a description in the materials and methods section of this revised version. The main difference between the method we used here and the method described by Dekens et al is that we used EdU instead of BrdU and we incubated the embryos with EdU on ice instead of injecting the compound.

We have now also included the data for EdU labeling on unfertilized eggs (activated squeezed eggs) in Fig.1 and summarize the results of the EdU labeling experiments in Table 2. Importantly, we never observed EdU incorporation in unfertilized eggs, confirming earlier described results by Dekens et al 2003.

Reviewer 1 suggested to perform EdU labeling experiments starting at 45 min to test whether arrested mutant embryos continue to cycle. We performed the suggested EdU labeling experiment and observed that 50% (22 out of 44) of the arrested MPcfap53-/- embryos continue to incorporate EdU even after 45 mpf and in the absence of cytokinesis. The experiment was performed with 4 independent lays from different pairs resulting in comparable results from the different lays. These results indicate that fertilized (EdU+) but arrested MPcfap53 mutant embryos fail to initiate karyokinesis and cytokinesis, but continue to replicate their DNA.

These results are now shown in Table 2b and described on p.7. of the revised manuscript.

4) The images for the developing MPcfap53-/- embryos should also be included with Figure 5D-I. Excluding the developing MPcfap53-/- from the staining in Figure 5D-I risks missing phenotypes that might differ from WT or phenotypes that might be similar to the arrested MPcfap53-/- embryos.

Response: We have now included images for developing MPcfap53 mutant embryos in figure 5 (panels J-L). The developing and arrested MPcfap53 embryos shown here are from the same clutch and were stained and processed in a similar manner. When analyzing these images we noticed that there are slight variations in these staining patterns, which were also observed in wild type embryos.

We added the following line to page 10:
‘ In developing MPcfap53-/- embryos we observed that γ-tubulin, PCM1 and Centrin accumulated at the MTOCs with slight variations in their patterns (Fig 5J-L), which may be due to natural variation observed among embryos.’

5) There is no gamma-tubulin staining in 5A-C but the authors state there is in the results section. This should be corrected in either the text or the image data. Also, PCM1 co-staining with Cfap53 should be performed since the authors are suggesting there is an interaction occurring between these proteins based on their MS data.

Response: We apologize for the confusion here. The gamma-tubulin and Cfap53 co-staining was shown in Figure 4 and not in Figure 5. In Figure 5 co-staining of GFP-Cfpa53 with Centrin is shown. We have corrected this in the text.
We agree that it would be helpful to show a co-localisation of PCM1 and Cfap53. We have tried several times but were unsuccessful. The signal of this staining was too weak to image and interpretate, which may be partly due to a new batch of anti-PCM1 antibody that we received. We are still working this out with the company that supplies the PCM1 antibody.

6) In some panels in Fig 5, the MTOCs are not positioned in line with the metaphase chromosomes, as normally is the case (e.g. D, E). This may be due to a mounting issue, if the ‘bridging’ method used to mount the cover slip applies pressure and squishes a bit the cells, causing displacement of the MTOC from the metaphase plate. This can also cause micronuclei to disperse as well and could in part cause the phenotype in G.

Response: We did not use the ‘bridging’ method for the embryo mounting, but instead mounted the embryos in agarose in a glass bottom dish and used an inverted microscope for the imaging (see description in Materials and Methods). We therefore don’t think that the positioning of the metaphase chromosomes or the dispersion of the micro nuclei are a consequence of applied pressure.

© 2022. Published by The Company of Biologists under the terms of the Creative Commons Attribution License (https://creativecommons.org/licenses/by/4.0/).

7) Were the embryos that were used for Figure 6 selected at random? There is an n = 6 shown for both the developing and arrested embryos, which does not agree with the ratios of developing to arrest embryos seen in other figures. Can the authors explain this possible discrepancy?

Response: The embryos used for Figure 6 were indeed selected at random from a clutch of embryos derived from cfap53 mutant parents. The ratio of developing and arrested embryos differed depending on the pair that was used. For the results of the different experiment described in the manuscript we have used different pairs of cfap53 mutants. All cfap53 mutant pairs showed the same phenotype of early arrest albeit with some variation in the penetrance of the phenotype. To illustrate this we have now included a table (Table 1) for which we scored developing and arrested embryos for different lays of 4 different cfap53 mutant pairs. We have also clarified that in the text (Results p.6).

8) The authors state that the GFP-Cfap53 transgene rescues left-right abnormalities and direct the reader to Figure S3, yet these data are not present in Figure S3. Please correct. The visual data would add to the quality of the rescue.

Response: The rescue of the laterality phenotype by the GFP-Cfpa53 was not included in Figure S3. We apologize for this mistake. We have now included these data in the revised manuscript (Figure S3B). Cardiac laterality was scored by visual inspection of the embryos followed by their genotyping. Please note that in the zygotic cfap53-/- embryos heart laterality is randomized, which results in half of the embryos having their heart positioned at the normal left side (left jog; scored as non-affected) while the other half have their hearts positioned at the midline (no-jog) or at the right side (right jog) (combined scored as affected) as we previously described in Noël et al. (2016), *Human mutation* 37, 194-200.

9) At least one analysis of gamma-tubulin, PCM1, or Centrin (similar to experiments in Figure 5D-I) should be performed on the MPcfap53-/- embryos that are rescued by the transgenic GFP-Cfap53 to show the extent of rescue.

Response: We have now performed analysis of gamma-tubulin with DAPI on MPcfap53-/- embryos that are rescued by GFP-Cfap53. These data are presented in Supplementary figure S5. We observed normal gamma-tubulin accumulation at both sides of the nuclear DNA and normal chromosome segregation indicating that GFP-Cfap53 rescued MTOC formation very efficiently.

10) The text in the results section (third sentence below “Cfap53 interactome” section title) says streptavidin-pulldown and cell culture work was done with both zebrafish Cfap53 and human CFAP53. I did not find evidence of human CFAP53 being used in either the Material and Methods section or the figures. This should be clarified or corrected.

Response: We apologize for the confusion about the use of human or zebrafish cfap53 for the pull down experiments. The pull down experiments followed by mass spectrometry were performed only with the human CFAP53 fused to bioGFP. We performed these experiments with both a N-terminal and a C-terminal fusion (bioGFP-CFAP53 and CFAP53-bioGFP). The description of these constructs can be found in the materials and method section under ‘Plasmid construction and transgenesis’. The zebrafish GFP-cfpa53 fusion was only used for the zebrafish experiments. In the original version of the manuscript we showed pictures of the zebrafish GFP-Cfpa53 protein localizing to the centrosomes in HeLa cells. This likely caused the confusion and we have therefore replaced the images with images of human bioGFP-CFAP53 and CFAP53-bioGFP costained with gamma-tubulin in HeLa cells.

11) Regarding analysis in figure 7, it is stated that the treatments do not effect g-Tubulin localization to the MTOC, but no secondary marker of the MTOC is examined. Either examine g-Tubulin with another MTOC marker or modify the conclusions.

Response: This sentence has been modified and now reads:
‘Importantly, nocodazole treatment of embryos during the third cell division, resulted in a cell cycle arrest at the 4-cell stage but had no apparent effect on gamma-tubulin localization compared to control treatment with DMSO only (Fig. 7E-F).’

© 2022. Published by The Company of Biologists under the terms of the Creative Commons Attribution License (https://creativecommons.org/licenses/by/4.0/).

Minor:
1) Authors state that "Cfap53 is required to initiate the first cell division," but saying required is a strong statement since some embryos do divide. Please reword. For example, the authors use "facilitates" in the title and in other areas in the results section.

Response: we agree with the reviewer that based on the results presented here, it can be concluded that Cfap53 facilitates the first cell division rather than being required. We have adjusted this on page 10.

2) The GFP-Cfap53 that is stated to be co-localizing with Centrin in Figure S4 (arrowhead) looks similar to the background GFP staining to the left of the arrowhead. Are there clearer images to make this point more convincing?

Response: The co-localization of GFP-Cfap53 with Centrin is indeed very weak, but was observed consistently in multiple images (30 out of 40 analysed). Unfortunately, there is no clearer image than the image provided in figure S4

3) What was used as an indicator for selecting arrested embryos at 30 mpf in Figure 6? Was there a clear distinction between the developing and arrested embryos at this time-point other than the staining results? Perhaps including an image of the 30 mpf embryos in Figure 1A would be helpful.

Response: At 30 mpf the MPcfap53 embryos all look very similar and the difference between developing and arrested embryos at this time point can only be observed by staining DNA and spindle components. From the time lapse experiment shown in Figure 1, the earliest time point we have is 45 mpf as it took this amount of time to mount the embryos and setup the timelapse procedure at the microscope.

4) Was immunolabeling for Cfap53 also performed for the nocadazole treatment experiments shown in Figure 7? If so, it would be valuable to include this data to show whether Cfap53 localization is also affected similar to gamma-tubulin (since the authors show that both proteins co-localize in Figure 4). Dispersion of Cfap53 due to destabilizing microtubules would agree with experiments performed in Silva et al. 2016. Further demonstrating the behavior and similarity of Cfap53 between different systems.

Response: We agree that this is an important question and we have therefore performed the nocodazole treatment on the GFP-Cfap53 embryos. Unfortunately, we were unable to draw any conclusions about the effect of nocodazole on GFP-Cfap53 localization as the signal at these early stages is very weak and variable, including in the controls.

5) Figure 2, indicate the number of males or MPcap53 male/female pairs tested? At least 2 different males and MP pairs should be tested, and 3 is best. Repeat if not two biological replicates and indicate the number of embryos tested for each.

Response: The number of males used in Fig.2A and B are indicated in the legend (7 males). For the other experiments shown in Fig.2 we used at least 2 different pairs. For panel C the number of embryos that were scored is reported in Table 2, for panels C and D it is indicated in figure. We also adjusted the text (p.6 and 7) to provide more details on biological repeats.

6) Figure 2A, B, the males should be just cfap53-/- rather than MPcfap53, correct?

Response: This is indeed a mistake in the figure and we like to thank the reviewer for pointing this out. This has now been corrected.

7) In Fig 2E, why is the nuclear morphology so different to Fig 1E. Please comment on this in the text.

Response: The nuclear morphology of the MPcfap53 embryos is variable and therefore the nuclei in Fig.1E and 2E look different. We have added this to the text on page 6:
'*While at 10 minutes post fertilization (mpf) and 20 mpf no differences were apparent, we*

© 2022. Published by The Company of Biologists under the terms of the Creative Commons Attribution License (https://creativecommons.org/licenses/by/4.0/).

observed that 50% (n=35) of the MPcfap53-/- embryos displayed an aberrant and variable nuclear morphology with dispersed chromosomes and micronuclei (Fig. 1D,E).'

8) Figure 4, it is a bit confusing to have a nucleus or nuclei depicted in the left schematics, when nuclei are not actually present at metaphase, anaphase stages. Please modify.

Response: We have removed the nuclei from the cartoons as these cartoons mainly have the purpose of illustrating the stage of the embryo and location of the images.

9) Page 6 "As unfertilized oocytes arrest in a similar manner,", once an oocyte passes through the oviduct, it is an egg. Change unfertilized oocytes to eggs. Same issue on p9, first paragraph, oocyte should be egg. And again on p15.

Response: We thank the reviewer for pointing this out and we have corrected this in the text throughout the manuscript.

10) On page 9, provide a reference for this statement during the cleavage stage: "Failure in MTOC formation will result in a disorganized microtubule network and cell cycle arrest, since proper bipolar spindles cannot be formed."

Response: A reference is now provided.

11) Provide N values for Fig S1, S3 S4 for adult tested (at least two needed), cells or sperm examined.

Response: Number are provided in the figures and legends.

12) Page 14, 1.000x change to 1,000x.

Response: Change was made.

Reviewer 3

Advance Summary and Potential Significance to Field:

The manuscript by Willekers et al documents the characterization of a maternal and paternal functions for the satellite protein Cfap52/Cdc11 in the zebrafish embryo. This gene has been previously shown to have effects zygotically in embryo laterality, and the authors now study an effect observable in the offspring of homozygous mutant embryos, revealing previously unknown functions in the gametes. The work also includes proteomic analysis to establish networks of protein interactions for this factor. The parental function of this gene is of interest and in addition the authors suggest a function for the gene specifically in the first mitotic cycle, which would be a novel type of function (see below, additionally including caveats for this interpretation).

Reviewer 3 Comments for the Author:

There are some concerns with the manuscript

Controls are not well documented. One set of controls involves the known fact that zebrafish fertilization rates during natural pair matings are known to vary widely from 0 to 100%. Thus, it is important to have clear and stringent protocols to address this potential variability, yet these are not well documented. It is unclear for example how many fish or crosses are used for the various experiments. Additionally, as the authors point out, embryos that lack a division in the first cell cycle are not readily distinguishable from unfertilized embryos. The primary strategy in this work to address this is to use a time course of fixed embryos, where the authors observe a normal fraction of pronuclei and pronuclear fusion in early stages – however there is not a direct correlation between the time course and the live phenotype (e.g. the same clutch(es) being used for both experiments). In the absence of a direct effort to address this, it is difficult to know whether the observed data is simply a consequence of clutch variability, rather than the authors' interpretation.

© 2022. Published by The Company of Biologists under the terms of the Creative Commons Attribution License (https://creativecommons.org/licenses/by/4.0/).

Response: The reviewers is asking for a better documentation of the number of crosses and pairs that were used to describe the MPcfap53-/- phenotype. We agree with the reviewer and therefore we included a table (Table1) that shows the number of affected embryos in matings from independent pairs. We also adjusted the description of the results on page 6 and 7 to include more information about the number of pairs that were tested.

In addition, we have now performed the experiment as suggested by the reviewer and analysed several clutches of independent pair matings for both nuclear congression and development (cytokinesis) (presented in Figure 2). For this purpose embryos from the same clutch were split in two groups, one group was fixed and stained with DAPI while the other group was analysed later for their development (normal cell divisions). The results are described on page 6 and read:
"To analyse whether nuclear congression is affected in MPcfap53-/- embryos we took clutches of MPcfap53-/- embryos and fixed a portion of the embryos at 15 mpf to analyse nuclear congression while the rest of the clutch was analysed live for cytokinesis. We analysed at least 3 independent wild type and cfap53-/- pairs and found no significant difference in nuclear congression between MPcfap53-/- and wild type embryos, while cytokinesis was affected in MPcfap53-/- (Fig. 2C)."

Another set of controls that are missing from the paper are generally, wild-type embryos (the authors rely on the unaffected mutant embryos for this) as well as unfertilized controls.

Response: We used wild type embryos as controls in Figures 1 (nuclear morphology) and 5 (gamma-tubulin, PCM1 and Centrin localisation). We now have included unfertilized eggs as control for the EdU labeling (see below).

Related to the last point, the authors should make a stronger case for the mutant arrested embryos not being unfertilized embryos. An example of the superficial way in which this is addressed (in addition to the time course of fixed embryos with technical caveats as stated above) is the fact that the authors use a DNA incorporation argument to support the idea that the mutants are not unfertilized embryos, since the authors see incorporation of EdU in mutant embryos but a previous study (Dekens et al 2002) had shown that unfertilized embryos do not. However, the Dekens et al used a different compound (BrdU) and it is unclear how comparable the methods used are (in particular the EdU protocol in this manuscript is missing in the Methods section). Even if the protocols were comparable, given the importance of the issue (unfertilized vs arrested) to the logical core of this study, the manuscript should include parallel studies on unfertilized embryos as controls/comparison.

Response: We apologize for omitting a detailed description of the EdU labeling experiment in the original manuscript. We now included a description in the materials and methods section of this revised version. Main difference between the method we used here and the method described by Dekens et al is that we used EdU instead of BrdU and we incubated the embryos with EdU on ice instead of injecting the compound.

To compare the EdU labeling method with the previously described method by Dekens et al, we incubated unfertilized (squeezed) eggs from wild type fish with EdU from 0-60 mpf. When we analyzed the EdU incorporation in unfertilized eggs we never observed EdU labeling (Table 2a; n=0/23, from 2 independent experiments). Results are presented in Figure 2 and the new Table 2. When we analyzed the EdU incorporation in wild type fertilized eggs we observed EdU labeling in all embryos that progressed to the 2-cell stage (n=23/23, from 2 independent experiments). Around 40% of the wild type embryos did not progress to the 2-cell stage and most (12 out of 15) did not incorporate EdU indicating that these were not fertilized. Some wild type embryos (3 out of 15) that did incorporate EdU but did not progress to the 2-cell stage are likely fertilized but arrested due to the incubation on ice. When we performed similar experiments with MZcfap53-/- we observed that a large proportion had not progressed into the 2-cell stage (55 out of 62, results from 3 independent experiments). From these, 29 had not incorporated EdU and are most likely non-fertilized eggs.
Importantly 26 embryos did incorporate EdU while these had not progressed to the 2-cell stage, indicated that these mutant embryos had been fertilized but arrested at the first cell division. As we observed EdU incorporation in 47% (n=26/55) of the arrested MZcfap53 mutant embryos and never observed EdU incorporation in unfertilized eggs we can conclude that these arrested MZcfap53 mutant embryos were fertilized and initiated DNA replication.

© 2022. Published by The Company of Biologists under the terms of the Creative Commons Attribution License (https://creativecommons.org/licenses/by/4.0/).

Reviewer 1 suggested to perform EdU labeling experiments starting at 45 min to test whether arrested mutant embryos continue to cycle. We performed the suggested EdU labeling experiment and observed again that 50% of the arrested MPcfap53-/- embryos incorporated EdU even after 45 mpf (Table 2b; *cfap53-/-* cross embryos at 1-cell stage, EdU+: n=22/44). In contrast, EdU incorporation in unfertilized oocytes was again 0% (n=0/26). The experiment was performed with 4 independent lays from different pairs of cfap53-/- fish with comparable results. These results indicate that when MZcfap53 mutant embryos are fertilized (based on EdU incorporation) they either develop normally or fail to undergo cytokinesis but continue DNA replication resulting in endoreduplication.

These results are now described on page 6-7:

The authors propose a model in which Cfap53 is required parentally only for the first mitotic division. This is a very unexpected discrete functional period for two reasons. First, centrosome and aster formation in the monoester involved pronuclear fusion are generally thought to be similar to those of the initial mitotic spindles, with the primary difference being the number of centriole pairs leading to a monoester vs bipolar conformation. If the first mitotic spindle but not the monoaster are affected, this would be to my knowledge the first study that shows functions specific for these two types of structures. The idea that the bipolar spindle is specifically affected is supported by the observation of Cfap53 appears localized during mitoses but not in the early embryo immediately after fertilization (however, this idea contrasts with the observation of Cfap53 in sperm). However, visualization of Cfap53 relies on a GFP construct, and there may be a lag in translation of maternal GFP protein after fertilization leading to the absence of labeling in the monoaster as a potential artefact. These potential caveats and complexities are not clearly addressed in the manuscript.

Response: We have added a few sentences to the discussion on page 13 for this:

"In earlier stages we only observed a diffuse GFP-Cfap53 signal, which either indicates a lack of specific localization or a lag in the translation of GFP-Cfap53 protein after fertilization. Cfpa53 is not required for nuclear congresion as this process was unaffected in MPcfap53-/- embryos. This is very different from the role of lymphoid-restricted membrane protein (lrmp), a maternally provided protein required for nuclear congression (Dekens et al., 2003; Lindeman and Pelegri, 2012). "

A second reason a discrete function only in the first mitosis is unexpected is that generally studies have shown that the mitoses in the early embryo are similar and part of a protracted egg-to-embryo transition that lasts into the cleavage stages. This proposed first-mitosis-only discrete function also conflicts with the observation of Cfap53 in the centrosomes in later stages of the cell cycle. Given this localization, a reasonable expectation would be that Cfap53 functions through the early cleavage early mitoses, not just the first one. Given that the arrest at the first cell cycle precludes observing the subsequent cycles, the authors seem to rely on several arguments to support a first-mitosis-only function. The first argument seems to be that the fraction of embryos that does not exhibit the arrest continue to divide normally – however, this can be explained by incomplete penetrance (see below) rather than temporal selectivity. A second argument is the differential effect of nocodazole treatment during the early period (0-45 min), which disrupts gamma-tubulin localization, compared to a later period (the third mitosis only), which does not. However, these treatments are of different length.
Additionally, the authors do not state a dechorionation step prior to drug treatment (Methods) and the chorion is known to be a partial barrier to nocodazole. Given that the chorion's structure and impermeability develops during egg activation, it could well be that the observed differential effect is due to the differential permeability of the chorion at early to later stages, in addition to the difference in time length of treatment.

Response: The nocodazole treatments for the early period (0-45 mpf) and late (60-85 mpf) are of different length because the cell cycles are of different length. Here we wanted the nocodazole treatment to last only during one cell division, since the treatment blocks cytokinesis and this may have secondary consequences. We did not dechorionate the embryos. We do not think that there is a difference in the permeability of the chorion for nocodazole as we did not see a difference in the response to nocodazole on cytokinesis, which was affected in both the early and late treatment.

© 2022. Published by The Company of Biologists under the terms of the Creative Commons Attribution License (https://creativecommons.org/licenses/by/4.0/).

Additionally, the observed fraction of embryos that are not affected by Cfap53 loss of function suggests that the function is not fully essential and that the embryos are in a functional threshold range leading to incomplete penetrance of the phenotype. Given this incomplete penetrance and near-threshold activity, one would also expect that the first cell division, which involves the largest cells and internal cellular structures, would be the most sensitive to functional reduction, leading to phenotypes that may be observable only in the first mitosis and not in subsequent mitosis. This is thought to be the case for example with the janus mutation, which preferentially affects the first cell division, likely because of sub-optimal cell-cell adhesion leading to a separation of the first (largest and needing greater cohesion) blastomeres.

Response: We agree with the reviewer that Cfap53 is not essential as there are always embryos that are not affected by the loss of Cfap53. We therefore tried to be careful with the conclusions and stated that Cfap53 facilitates the formation of the first MTOC. We also agree that the large size of the cell during the first cell division may explain why the loss of Cfap53 is affecting only the first division and not those following. It may well be that due to the large cell size passive diffusion of MTOC components is not always sufficient and also requires active transport via microtubules. This would be in line with the results from the nocodazole experiments. In subsequent cell divisions, due to the smaller cell size passive diffusion may be sufficient. We discuss this on page 13 and 14.

Regarding the paternal effect, there is no control for the males' mating behavior. Zebrafish fertilization occurs in a tight window where sperm ejection needs to be in close proximity of the eggs, with sperm entry needed before the post activation sperm blocks occurs. Thus any changes in behavior could increase the fraction of unfertilized eggs, which given uncertainties as stated above could be confused with arrested embryos. Given that the males have laterality defects, it is possible that they could have defects in mating behavior. This could be controlled using in vitro fertilization, though in this case sperm function and concentration would have to be comparable.

Response: We cannot exclude that fertlization rates are affected by different behavior of the cfap53 mutant males or females and we do have some indication from the EdU incorporation assay that fertilization rates may also contribute to the observed phenotype (albeit in addition to Cfap53 promoting the first cell division(s)). In the revised version we have more data using the EdU incorporation assay (Table2). We noticed that approximately 50% of the arrested MPcfap53-/- embryos were EdU negative (Table2a; 1-cell stage embryos from cfap53-/- cross, EdU-: n=29/55, Table 2b; 1-cell stage embryos from cfap53-/- cross, EdU-: n=22/44). These either represent unfertilized eggs or fertilized embryos that did not take up the EdU efficiently enough to detect it. That this can happen is evident as the efficiency of the EdU incorporation in developing wild type embryos ranged between 100% and 77% (Table 2a; 2-cell stage embryos from wild type cross, EdU+: n=23/23, Table 2b; 32-cell stage embryos from wild type cross, EdU+: n=40/52).

We have added a sentence in the results section (p. 6-7) to address this:
"In crosses from cfap53-/- pairs we found that 53% of the arrested embryos had not incorporated the EdU indicating that these were not fertilized or did not take up the EdU efficiently (Table 2a; cfap53-/- cross embryos at 1-cell stage, EdU-: n=29/55)"

We do not expect that by using in vitro fertilization this can be solved as fertilization rates in in vitro fertlization experiments are too variable and preclude better assessment of these small differences.
In the Discussion: "Why these structures that function as MTOCs are so large is not clear, but might have to do with the large size of the embryo (see also below)". There is extensive literature documenting that given the limited maternal pools for products needed for cell division, cellular structures are large at first and become smaller gradually as they adjust to smaller cell sizes (cellular scaling, see for example literature by M. Wühr).

Response: we thank the reviewer for pointing this out and have modified the discussion on page 13 and included some references to illustrate scaling of MTOC size in embryos.

© 2022. Published by The Company of Biologists under the terms of the Creative Commons Attribution License (https://creativecommons.org/licenses/by/4.0/).

Second decision letter

MS ID#: DEVELOP/2020/198762

MS TITLE: The centriolar satellite protein Cfap53/Ccdc11 facilitates the formation of the first zygotic microtubule organizing center in the zebrafish embryo

AUTHORS: Sven Willekers, Federico Tessadori, Babet van der Vaart, Heiko H.W. Henning, Riccardo Stucchi, Maarten Altelaar, Bernard A.J. Roelen, Anna Akhmanova, and Jeroen Bakkers

I have now received all the referees' reports on the above manuscript, and have reached a decision. The referees' comments are appended below, or you can access them online: please go to BenchPress and click on the 'Manuscripts with Decisions' queue in the Author Area.

As you will see, there is a difference in opinion between the referees regarding the suitability of your study for publication inDevelopment. Although referees 1 and 2 are happy with your revisions, referee 3 is not. Given that some of the criticisms raised by this reviewer relate to the robustness of your conclusions and interpretations, it is important that you respond to the points that he/she makes.

Please attend to all of the reviewers' comments and ensure that you clearly highlight all changes made in the revised manuscript. Please avoid using 'Tracked changes' in Word files as these are lost in PDF conversion. I should be grateful if you would also provide a point-by-point response detailing how you have dealt with the points raised by the reviewers in the 'Response to Reviewers' box. If you do not agree with any of their criticisms or suggestions please explain clearly why this is so.

Reviewer 1

Advance summary and potential significance to field

The authors identify a requirement for maternal and paternal cfap53 in formation of the MTOC in the early zebrafish embryo. This identifies unique aspects of the first division of the zygote and support for essential paternal contributions to this process.

Comments for the author

The authors have addressed my concerns

Reviewer 2

Advance summary and potential significance to field

The authors present work that describes a novel role for cfap53 in establishing a functional MTOC during the first cell division in developing zebrafish embryos. They found that the loss of maternal and paternal cfap53 leads to embryos that fail to undergo the first cell division due to a defect in MTOC formation in maternal-paternal cfap53 loss-of-function mutants. The first mitotic division is a critical stage in embryonic development and understanding how this process is faithfully executed is of broad interest to the developmental biology community and of particular interest to researchers that study early embryonic development. This makes this new role for cfap53 of interest to a broad audience of Development readers.

Comments for the author

The authors have addressed all of the previous points and this study is ready for publication in Development. Just one minor point, regarding the results shown in Figure 7 and conclusions. Might it be that once the centrosomes/MTOC's have formed during that first cell cycle that they are no longer dependent on microtubules for assembly. That is, they have formed and do not dissociate between mitoses. While duplication of the centrosome/MTOC could depend on microtubules, it

© 2022. Published by The Company of Biologists under the terms of the Creative Commons Attribution License (https://creativecommons.org/licenses/by/4.0/).

isn't clear that was tested in the experiment, since the cell cycle arrests with nocodozole treatment. It need not be tested, just thinking about the conclusion made. I leave it to the authors if this is worth addressing. I do like in the Discussion the integration of these results with what is known about how MTOC proteins are either delivered via microtubules or associate with it via diffusion.
Fig 7, not sure if 'cel' in the figure is a typo.

Reviewer 3

Advance summary and potential significance to field

The manuscript by Willekers et al documents the characterization of a maternal and paternal functions for the satellite protein Cfap52/Cdc11 in the zebrafish embryo.
This gene has been previously shown to have effects zygotically in embryo laterality, and the authors now study an effect observable in the offspring of homozygous mutant embryos, revealing previously unknown functions in the gametes.
The work also includes proteomic analysis to establish networks of protein interactions for this factor. The parental function of this gene is of interest and in addition the authors suggest a function for the gene specifically in the first mitotic cycle, which would be a novel type of function (see below additionally including caveats for this interpretation).

Comments for the author

Comments on revision
The authors have carried out an effort to address previous concerns. For example carrying out the experiment in the same clutches better substantiates that cytokinesis seems to be significantly affected, whereas pronuclear fusion does not, showing a role for Cfap53 specific to cell division. This in itself is an interesting observation although the idea that maternal stores for genes coding for centriolar components are needed for early embryonic cytokinesis is not surprising. One aspect of this work that makes it remarkable is the idea that Cfap53 (maternal and paternal) is involved specifically in MTOCs for the first mitotic division
In my previous comments I tried to convey concerns on why this model does not fit well our biological understanding of early development, seeking better substantiation for such a quite unexpected first-cycle-specific function. While there has been improvement in the manuscript, the response and revisions do not completely alleviate these concerns.
A paternal role for Cfap53: a role for maternal components is well documented and therefore the phenotype of MZ and M mutant embryos is what one would expect given a maternal requirement for centriolar components. There is also a precedent for paternal effects, in particular it would not be unreasonable to think that centriolar-associated factors in the sperm can have an effect in early embryos.
However, given that pronuclear fusion is not affected, these centriolar factors would have to be dedicated to the mitotic spindle and not just the interphase monoaster/sperm aster dedicated to pronuclear fusion. This (to act in MTOC during mitosis but not earlier) could be a novel role for a sperm factor, hence whether the paternal genotype matters is quite significant. The authors have tried to corroborate the paternal function in offspring of mutant males and wild type females by carrying out "at least three different matings with the same fish pair"
(Figure legend 1). This means all experiments appear to have been made with the same male! Additionally the "arrested" category can not distinguish in this experiment unfertilized vs embryos stalled in mitosis, and we also know the male may have laterality defects and therefore potentially exhibit reduced fertilization rates in natural matings. In their rebuttal, the authors acknowledge that they can not exclude behavior as a variable and that indeed some of their own evidence suggests fertilization rates are contributing to the phenotype.
Altogether, considering a single tested male, lack of direct observation of the cellular phenotype and other potential uncontrolled variables, the idea that there is a paternal function does not seem as presented properly substantiated.

On the apparent specificity of function for the first cell division: In their rebuttal, the authors referring to a previous concern in interpretation appear to "agree that the large size of the cell during the first cell division may explain why the loss of Cfap53 is affecting only the first division".

© 2022. Published by The Company of Biologists under the terms of the Creative Commons Attribution License (https://creativecommons.org/licenses/by/4.0/).

However, the emphasis throughout the manuscript (in the title "formation of the first zygotic MTOC"; abstract "Once the zygotic MTOC is formed, Cfap53 is dispensable for MTOC formation and integrity in subsequent cell divisions"; first sentence of Discussion "The first cell division in embryogenesis is different compared to following cell divisions, as it involves the formation of the first MTOC from maternal and paternal components") seems to highlight a first-cycle-specific function. An incompletely penetrant loss of function phenotype can show an apparently specific phenotype in an event that requires the highest threshold of activity (in this case the first mitotic cell cycle with the highest threshold but not later divisions with lower thresholds), but that does not mean that the function is "dispensable" for subsequent cell divisions. Indeed, Cfap53 appears to colocalize with g-tubulin at the 8-cell stage in Figure 4, indicating a likely function for Cfap53 during this "later" cell cycle.
(Incidentally, the sentence in the Discussion "The first cell division in embryogenesis is different compared to following cell divisions, as it involves the formation of the first MTOC from maternal and paternal components" is not correct, since the first MTOC reconstituted from maternal and paternal components is that of the sperm aster/monoaster)
The authors also state in the rebuttal that they do not believe that the different length of nocodazole treatment is relevant to the different results in early and late embryos with regards to g-tubulin localization, because both treatments affect cytokinesis. This is not a convincing argument since cytokinesis as a whole and g-tubulin localization specifically could be affected by different thresholds of drug treatment. This argumentation seems to mirror the rest of the manuscript in arguing that the first division is different from the others from a perspective of Cfap53 function, which seems a broad over-interpretation.
As presented, the authors conclusively show a role for Cfap53 in the early embryo (likely maternal) on mitotic MTOC spindle formation in the early embryo, but the claim that the function is i) paternally provided or ii) specific to the very first mitosis (as opposed to later mitoses) remain insufficiently substantiated.
What is better substantiated is that the MTOCs in the early mitoses require Cfap53 function, whereas those for the sperm aster/monoaster do not. If this is indeed the case, to my knowledge this may be the first time that MTOCs are differentiated, involving differential functional requirements, from the sperm aster/monoaster to the bipolar spindle stage.

Minor issues
Page 11: cao/sas-6 (typo, it is cea/sas-6)
Page 14: the comparison to lrmp is In figure 2d, the close ups of the pronuclei are nice but in the bottom panel for example it is not possible to tell whether the other pronucleus is indeed present. A lower mag as in 1C, D would with magnified inserts would show this.
Figure 7: typos "nocadazole"

Second revision

Author response to reviewers' comments

Response to reviewers.
We want to thank all reviewers for their time to carefully read the manuscript and providing constructive suggestion on how to improve the quality of the manuscript. We have now addressed all remaining concerns in a point-to-point response.
Reviewer 1

Advance Summary and Potential Significance to Field:
The authors identify a requirement for maternal and paternal cfap53 in formation of the MTOC in the early zebrafish embryo. This identifies unique aspects of the first division of the zygote and support for essential paternal contributions to this process.

Reviewer 1 Comments for the Author:
The authors have addressed my concerns

© 2022. Published by The Company of Biologists under the terms of the Creative Commons Attribution License (https://creativecommons.org/licenses/by/4.0/).

Reviewer 2
Advance Summary and Potential Significance to Field:
The authors present work that describes a novel role for cfap53 in establishing a functional MTOC during the first cell division in developing zebrafish embryos. They found that the loss of maternal and paternal cfap53 leads to embryos that fail to undergo the first cell division due to a defect in MTOC formation in maternal-paternal cfap53 loss-of-function mutants. The first mitotic division is a critical stage in embryonic development and understanding how this process is faithfully executed is of broad interest to the developmental biology community and of particular interest to researchers that study early embryonic development. This makes this new role for cfap53 of interest to a broad audience of Development readers.

Reviewer 2 Comments for the Author:
The authors have addressed all of the previous points and this study is ready for publication in Development. Just one minor point, regarding the results shown in Figure 7 and conclusions. Might it be that once the centrosomes/MTOC's have formed during that first cell cycle that they are no longer dependent on microtubules for assembly. That is, they have formed and do not dissociate between mitoses. While duplication of the centrosome/MTOC could depend on microtubules, it isn't clear that was tested in the experiment, since the cell cycle arrests with nocodozole treatment. It need not be tested, just thinking about the conclusion made. I leave it to the authors if this is worth addressing. I do like in the Discussion the integration of these results with what is known about how MTOC proteins are either delivered via microtubules or associate with it via diffusion.

Response: Based on the comments from reviewer 3 we have decided to remove the second part of figure 7 (nocodazole treatment after first cell division). It is indeed an interesting suggestion made by the reviewer and we have also considered this. However, more extensive experiments would be required to study this further and this would go beyond the scope of this manuscript.

Fig 7, not sure if 'cel' in the figure is a typo.

Response: This was indeed a typo and has been corrected.

Reviewer 3

Advance Summary and Potential Significance to Field:
The manuscript by Willekers et al documents the characterization of a maternal and paternal functions for the satellite protein Cfap52/Cdc11 in the zebrafish embryo.
This gene has been previously shown to have effects zygotically in embryo laterality, and the authors now study an effect observable in the offspring of homozygous mutant embryos, revealing previously unknown functions in the gametes.
The work also includes proteomic analysis to establish networks of protein interactions for this factor. The parental function of this gene is of interest and in addition the authors suggest a function for the gene specifically in the first mitotic cycle, which would be a novel type of function (see below, additionally including caveats for this interpretation).

Reviewer 3 Comments for the Author:
Comments on revision

The authors have carried out an effort to address previous concerns. For example, carrying out the experiment in the same clutches better substantiates that cytokinesis seems to be significantly affected, whereas pronuclear fusion does not, showing a role for Cfap53 specific to cell division. This in itself is an interesting observation although the idea that maternal stores for genes coding for centriolar components are needed for early embryonic cytokinesis is not surprising. One aspect of this work that makes it remarkable is the idea that Cfap53 (maternal and paternal) is involved specifically in MTOCs for the first mitotic division

In my previous comments I tried to convey concerns on why this model does not fit well our biological understanding of early development, seeking better substantiation for such a quite

© 2022. Published by The Company of Biologists under the terms of the Creative Commons Attribution License (https://creativecommons.org/licenses/by/4.0/).

unexpected first-cycle-specific function. While there has been improvement in the manuscript, the response and revisions do not completely alleviate these concerns.

A paternal role for Cfap53: a role for maternal components is well documented and therefore the phenotype of MZ and M mutant embryos is what one would expect given a maternal requirement for centriolar components. There is also a precedent for paternal effects, in particular it would not be unreasonable to think that centriolar-associated factors in the sperm can have an effect in early embryos. However, given that pronuclear fusion is not affected, these centriolar factors would have to be dedicated to the mitotic spindle and not just the interphase monoaster/sperm aster dedicated to pronuclear fusion. This (to act in MTOC during mitosis but not earlier) could be a novel role for a sperm factor, hence whether the paternal genotype matters is quite significant. The authors have tried to corroborate the paternal function in offspring of mutant males and wild type females by carrying out “at least three different matings with the same fish pair” (Figure legend 1). This means all experiments appear to have been made with the same male! Additionally the “arrested” category can not distinguish in this experiment unfertilized vs embryos stalled in mitosis, and we also know the male may have laterality defects and therefore potentially exhibit reduced fertilization rates in natural matings. In their rebuttal, the authors acknowledge that they can not exclude behavior as a variable and that indeed some of their own evidence suggests fertilization rates are contributing to the phenotype.
Altogether, considering a single tested male, lack of direct observation of the cellular phenotype and other potential uncontrolled variables, the idea that there is a paternal function does not seem as presented properly substantiated.

Response: We agree with the reviewer that the paternal versus maternal requirement for Cfap53 was not worked out sufficiently to draw clear conclusions. We therefore have repeated these experiments with additional mutant male and mutant female fish crossed to wild type fish and included these results in Table 1. We tested 5 mutant males and 5 mutant females and the new results confirm our previous conclusion that there is both a maternal as well as a paternal effect for the loss of Cfap53. We have removed panel 1E from the manuscript as it was redundant with the data provided in Table 1.

To distinguish whether the cell division arrest observed in embryos from mutant males or mutant females was due to reduced fertility or due to a cell division arrest we also repeated the EdU labeling experiments with such embryos. Importantly, embryos derived from either male cfap53-/- or female cfap53-/- crossed to wild type fish incorporated the EdU in the arrested embryos. These results are shown in Table 2. The percentage of EdU incorporation was highest in the embryos derived from cfap53-/- males (88%) compared to cfap53-/- females (55%) and indicates that arrested Pcfap53-/- embryos were mostly fertilized but failed to progress through a cell division. These new results clearly demonstrate a paternal function for Cfap53.

On the apparent specificity of function for the first cell division: In their rebuttal, the authors referring to a previous concern in interpretation appear to “agree that the large size of the cell during the first cell division may explain why the loss of Cfap53 is affecting only the first division”. However, the emphasis throughout the manuscript (in the title “formation of the first zygotic MTOC”; abstract “Once the zygotic MTOC is formed, Cfap53 is dispensable for MTOC formation and integrity in subsequent cell divisions”; first sentence of Discussion “The first cell division in embryogenesis is different compared to following cell divisions, as it involves the formation of the first MTOC from maternal and paternal components”) seems to highlight a first-cycle-specific function. An incompletely penetrant loss of function phenotype can show an apparently specific phenotype in an event that requires the highest threshold of activity (in this case the first mitotic cell cycle with the highest threshold but not later divisions with lower thresholds), but that does not mean that the function is “dispensable” for subsequent cell divisions. Indeed, Cfap53 appears to colocalize with g-tubulin at the 8-cell stage in Figure 4, indicating a likely function for Cfap53 during this “later” cell cycle.

Response: Since we don’t have additional proof for this ‘first cell division’ specific role and obtaining such evidence would require substantial additional experiments we toned down this claim. We have made textual changes to the different sections of the manuscript by removing specific reference to the first cell division. These textual changes are marked in blue text.

© 2022. Published by The Company of Biologists under the terms of the Creative Commons Attribution License (https://creativecommons.org/licenses/by/4.0/).

(Incidentally, the sentence in the Discussion “The first cell division in embryogenesis is different compared to following cell divisions, as it involves the formation of the first MTOC from maternal and paternal components” is not correct, since the first MTOC reconstituted from maternal and paternal components is that of the sperm aster/monoaster)

Response: This sentence was removed

The authors also state in the rebuttal that they do not believe that the different length of nocodazole treatment is relevant to the different results in early and late embryos with regards to g-tubulin localization, because both treatments affect cytokinesis. This is not a convincing argument since cytokinesis as a whole and g-tubulin localization specifically could be affected by different thresholds of drug treatment. This argumentation seems to mirror the rest of the manuscript in arguing that the first division is different from the others from a perspective of Cfap53 function, which seems a broad over-interpretation.

Response: Since we don’t have any addition evidence to strengthen our conclusion (see above) we have removed these data (Fig. 7E and F) from the manuscript as well as the conclusion that the first cell division is different from the subsequent divisions.

As presented, the authors conclusively show a role for Cfap53 in the early embryo (likely maternal) on mitotic MTOC spindle formation in the early embryo, but the claim that the function is i) paternally provided or ii) specific to the very first mitosis (as opposed to later mitoses) remain insufficiently substantiated. What is better substantiated is that the MTOCs in the early mitoses require Cfap53 function, whereas those for the sperm aster/monoaster do not. If this is indeed the case, to my knowledge this may be the first time that MTOCs are differentiated, involving differential functional requirements, from the sperm aster/monoaster to the bipolar spindle stage.

Response: Concerning the first point, in this second revised version of the manuscript we have provided additional evidence to strengthen our conclusion that Cfap53 function is also paternally provided. Concerning the second point, we have now removed the conclusion that Cfap53 is specifically required for the first cell division and instead focused more on the difference between MTOCs for the sperm aster and mitotic spindle.

Minor issues

Page 11: cao/sas-6 (typo, it is cea/sas-6)

Response: This typo has been corrected

Page 14: the comparison to lrmp is

Response: We were unable to responded to this comment as the sentence was incomplete.

In figure 2d, the close ups of the pronuclei are nice but in the bottom panel for example it is not possible to tell whether the other pronucleus is indeed present.
A lower mag as in 1C, D would with magnified inserts would show this.
Response: We did not have the lower magnification image for this embryo with the second nucleus. We therefore replaced the image with another example in which the two separated pronuclei are clearly visible.

Figure 7: typos “nocadazole”

Response: We corrected the typo

© 2022. Published by The Company of Biologists under the terms of the Creative Commons Attribution License (https://creativecommons.org/licenses/by/4.0/).

Third decision letter

MS ID#: DEVELOP/2020/198762

MS TITLE: The centriolar satellite protein Cfap53/Ccdc11 facilitates the formation of the zygotic microtubule organizing center in the zebrafish embryo

AUTHORS: Sven Willekers, Federico Tessadori, Babet van der Vaart, Heiko H.W. Henning, Riccardo Stucchi, Maarten Altelaar, Bernard A.J. Roelen, Anna Akhmanova, and Jeroen Bakkers

I sent your manuscript back to the one remaining critical referee and have now obtained their report. As you will see they still have quite a few comments but are much happier with this version. I think that their comments are constructive and may help you further improve the manuscript prior to publication. I will not be sending the manuscript back to the referee again and so any further changes you make are at your discretion.. The referee's comments are appended below, or you can access them online: please go to BenchPress and click on the 'Manuscripts with Decisions' queue in the Author Area.

Reviewer 3

Advance summary and potential significance to field

This is an improved revised manuscript and both better substantiates the results and conveys their significance. A major aspect of the significance in terms of novelty and developmental mechanism is the selective nature of the mutation on the mitotic spindle and many of my comments aim at further improving describing this aspect of the work.

Comments for the author

The authors use EdU incorporation to control for potential differences in fertilization rates, which is a helpful to control for such variability. The statement that females showed atypical mating behavior, with egg release “much later” is vague and unquantified, and it is unclear how many individuals were studied and whether this simply reflects random variation. (A lower fertilization rate when a maternal factor compared to when a paternal factor is missing is also predicted by the much larger size of the egg and presumably larger maternal contribution vs paternal contribution, hence behavior need not be invoked to explain this difference). In the absence of more detailed studies and since the authors are already controlling for fertilization rates using EdU, the statement on mating behavior would be better off deleted, or modified to acknowledge that mating behavior could be another contributing factor that has not yet been rigorously addressed.
The authors test the effect of MT depolymerization on g-tubulin, but not on Cfap53 itself. This omission is puzzling considering the main emphasis of the work.
Showing that Cfap53 protein localization to centriole does not depend on MT localization would help provide support for a role on centrosome formation, for example through phase separation
The authors make statements such as “Cfap53 is dispensable for sperm aster assembly” (abstract) and has a role that “is dispensable for sperm aster formation” (summary at the beginning of the Discussion). However, the sperm aster itself (e.g. detecting microtubules) has not been directly visualized in this study, and instead a normal function for the sperm aster is inferred by normal pronuclear congression. In the opinion of this Reviewer, not examining the sperm aster directly is a relatively simple experiment and a missed opportunity in this study (given that specificity for the mitotic spindle is an important aspect of the novelty on the work). If the authors choose to base their conclusions on the data as now shown, the conclusions should concordantly reflect this by toning down the statements, such as stating that Cfap53 function “appears to be dispensable for sperm aster assembly, as assessed by normal pronuclear congression”, or similar.
There has been significant improvement in the manuscript on describing and documenting a role specific to the mitotic cycles, as opposed to the sperm aster.
One aspect that is still not clearly conveyed is how this could occur. The authors statements remain very vague (from the Discussion, “different roles…forming the sperm aster and the mitotic spindle may be related to their difference in composition and size”. Clearly a mitotic spindle and a sperm aster have common elements (MTOCs nucleating radial aster microtubules) and differences

© 2022. Published by The Company of Biologists under the terms of the Creative Commons Attribution License (https://creativecommons.org/licenses/by/4.0/).

(a single MTOC vs two, plus additional components to nucleate other types of microtubules involved in mitosis). This basic context could be more clearly conveyed. Also important is how mechanistically would the maternal and paternal function be required for the first mitosis. For the maternal contribution this is relatively straightforward (maternal stores plus specificity of a function in the bipolar spindle), but not so clear for the paternal contribution. How is Cfap53 transmitted from the sperm so that it acts at the first mitotic spindle? The sperm is known to carry a pair of centrioles (or possibly one complete and another one needing completion), each of which will act as the main nucleator for the MTOCs in the first mitotic cell cycle - could paternal Cfap53 remain associated with each of the sperm-derived centrioles, which explains their effect later during the first mitosis? It would be helpful if the work at least discussed such mechanistic aspects, which are crucial to understand the basis of the observed phenomena.
EdU incorporation in Table 2 appear to show absolute numbers of embryos but it would be more helpful if presented as percentages, so that it can be more readily compared to the cell division phenotype in Table 1. To simplify the table, the two different trials in Table 2 could be pooled if they show similar effects.

Other issues:
The abbreviation MPcfap53-/- is defined, but not those for Mcfap53-/- or Pcfap53-/-}
On the statement regarding mutant embryos exhibiting endoreduplication, the Dekens et al studies on futile cycle should be added as a previous reference on this phenomenon and added context
In the Discussion, the authors invoke transcriptional adaptation to explain the variability of the phenotype. There are other reasons why the phenotype could be variable without transcriptional adaptation, in particular given the role of Cfap53 in the formation of a subcellular organelle that is known to be driven by phase separation, and where gene products may be adding accuracy in the formation of such organelles. Our knowledge of gene function is heavily biased towards genes that have 100% effects when mutated, however it is likely many genes have more subtle defects but which nevertheless are still selected by evolutionary processes. The high complexity domains in the protein further support a role in phase separation, providing further support to the idea that the product could be involved in enhancing the efficiency of a phase-separated process. The manuscript seems to underemphasize the potential for Cfap53 in phase separation – even if experiments do not directly address this, centrosomes are known to be phase separated structures and given the domains in Cfap53 it would be reasonable to at least highlight even if in the Discussion the potential of the protein to contribute to this process. (Incidentally, in the Discussion "a passive process called liquid-liquid phase separation": LLPS is now well established and suggest deleting "called" here)

In the Discussion, the reference to cea/sas-6 depending on the maternal and paternal phenotype is stated as Yabe et al 2007. It would be appropriate to also include a reference to previous work by O'Connell and White on C. elegans on Sas-6, which was the first to show such maternal and paternal contribution for centriolar components

---

Third revision

Author response to reviewers' comments

Reviewer 3 Advance Summary and Potential Significance to Field:
This is an improved revised manuscript and both better substantiates the results and conveys their significance. A major aspect of the significance in terms of novelty and developmental mechanism is the selective nature of the mutation on the mitotic spindle and many of my comments aim at further improving describing this aspect of the work.

Reviewer 3 Comments for the Author:
The authors use EdU incorporation to control for potential differences in fertilization rates, which is a helpful to control for such variability. The statement that females showed atypical mating behavior, with egg release "much later" is vague and unquantified, and it is unclear how many individuals were studied and whether this simply reflects random variation. (A lower fertilization rate when a maternal factor compared to when a paternal factor is missing is also predicted by the

© 2022. Published by The Company of Biologists under the terms of the Creative Commons Attribution License (https://creativecommons.org/licenses/by/4.0/).

much larger size of the egg and presumably larger maternal contribution vs paternal contribution, hence behavior need not be invoked to explain this difference). In the absence of more detailed studies and since the authors are already controlling for fertilization rates using EdU, the statement on mating behavior would be better off deleted, or modified to acknowledge that mating behavior could be another contributing factor that has not yet been rigorously addressed.

Response: We agree with this comment and have deleted the statement on mating behavior from the manuscript (page 7).

The authors test the effect of MT depolymerization on g-tubulin, but not on Cfap53 itself. This omission is puzzling considering the main emphasis of the work.
Showing that Cfap53 protein localization to centriole does not depend on MT localization would help provide support for a role on centrosome formation, for example through phase separation

Response: We have tried to test the effect of MT depolymerization on Cfap53 localization but we were unable to draw any conclusions because of the technical limitations to detect Cfap53 protein at these very early stages.
The Cfap53 signal was too weak to reliably and quantitively measure differences between control and nocodazole treated embryos.

The authors make statements such as “Cfap53 is dispensable for sperm aster assembly” (abstract) and has a role that “is dispensable for sperm aster formation” (summary at the beginning of the Discussion). However, the sperm aster itself (e.g. detecting microtubules) has not been directly visualized in this study, and instead a normal function for the sperm aster is inferred by normal pronuclear congression. In the opinion of this Reviewer, not examining the sperm aster directly is a relatively simple experiment and a missed opportunity in this study (given that specificity for the mitotic spindle is an important aspect of the novelty on the work). If the authors choose to base their conclusions on the data as now shown, the conclusions should concordantly reflect this by toning down the statements, such as stating that Cfap53 function “appears to be dispensable for sperm aster assembly, as assessed by normal pronuclear congression”, or similar.

Response: We have made textual changes to make clear that the role of Cfpa53 in sperm aster formation and function was assessed by investigating nuclear congression (changes in blue text).

There has been significant improvement in the manuscript on describing and documenting a role specific to the mitotic cycles, as opposed to the sperm aster. One aspect that is still not clearly conveyed is how this could occur. The authors statements remain very vague (from the Discussion, “different roles…forming the sperm aster and the mitotic spindle may be related to their difference in composition and size”. Clearly a mitotic spindle and a sperm aster have common elements (MTOCs nucleating radial aster microtubules) and differences (a single MTOC vs two, plus additional components to nucleate other types of microtubules involved in mitosis). This basic context could be more clearly conveyed. Also important is how mechanistically would the maternal and paternal function be required for the first mitosis. For the maternal contribution this is relatively straightforward (maternal stores plus specificity of a function in the bipolar spindle), but not so clear for the paternal contribution. How is Cfap53 transmitted from the sperm so that it acts at the first mitotic spindle? The sperm is known to carry a pair of centrioles (or possibly one complete and another one needing completion), each of which will act as the main nucleator for the MTOCs in the first mitotic cell cycle - could paternal Cfap53 remain associated with each of the sperm-derived centrioles, which explains their effect later during the first mitosis? It would be helpful if the work at least discussed such mechanistic aspects, which are crucial to understand the basis of the observed phenomena.

Response: These are all very relevant questions, but cannot be addressed without performing addition experiments. We hope that our results will spur more research to address these questions in the future. We have added some comments to the discussion to highlight these remaining questions.

EdU incorporation in Table 2 appear to show absolute numbers of embryos but it would be more helpful if presented as percentages, so that it can be more readily compared to the cell division

© 2022. Published by The Company of Biologists under the terms of the Creative Commons Attribution License (https://creativecommons.org/licenses/by/4.0/).

phenotype in Table 1. To simplify the table, the two different trials in Table 2 could be pooled if they show similar effects.

Response: Parts a and b of Table2 are the results of two different experiments (different exposure times for the EdU) and therefore cannot be pooled together. For better comparison with Table1 we now have included the percentages (between brackets) to Table 2.

Other issues:
The abbreviation MPcfap53-/- is defined, but not those for Mcfap53-/- or Pcfap53-/-}

Response: we have now included the description of these abbreviations on page 7.

On the statement regarding mutant embryos exhibiting endoreduplication, the Dekens et al studies on futile cycle should be added as a previous reference on this phenomenon and added context

Response: we have included the reference here.

In the Discussion, the authors invoke transcriptional adaptation to explain the variability of the phenotype. There are other reasons why the phenotype could be variable without transcriptional adaptation, in particular given the role of Cfap53 in the formation of a subcellular organelle that is known to be driven by phase separation, and where gene products may be adding accuracy in the formation of such organelles. Our knowledge of gene function is heavily biased towards genes that have 100% effects when mutated, however it is likely many genes have more subtle defects but which nevertheless are still selected by evolutionary processes. The high complexity domains in the protein further support a role in phase separation, providing further support to the idea that the product could be involved in enhancing the efficiency of a phase-separated process. The manuscript seems to underemphasize the potential for Cfap53 in phase separation – even if experiments do not directly address this, centrosomes are known to be phase separated structures and given the domains in Cfap53 it would be reasonable to at least highlight even if in the Discussion the potential of the protein to contribute to this process. (Incidentally, in the Discussion "a passive process called liquid-liquid phase separation": LLPS is now well established and suggest deleting "called" here)

Response: We agree that besides transcriptional adaptation, there are other reasons why the phenotype could be variable. We acknowledge this by stating: 'The cytokinesis defect that we observed in MP*cfap53-/-* embryos has an incomplete penetrance (see Table 1). This suggests that the loss of Cfap53 can be compensated by other mechanisms (see below discussion about diffusion versus active protein transport) or by proteins with a similar function.'
A possible role of Cfap53 in phase separation is also highlighted in the discussion: 'It is possible that similarly to the C. elegans centrosome component, the coiled-coil proteins SPD-5, CFAP53 facilitates MTOC formation by passive liquid-liquid phase separation (Woodruff, 2018; Woodruff et al., 2017).'
We do not wish to emphasize this further as we observed that active MT-based transport is required for γ-tubulin localization during the first division, which suggests that besides liquid-liquid phase separation, other mechanisms are important during MTOC formation.
In the Discussion, the reference to cea/sas-6 depending on the maternal and paternal phenotype is stated as Yabe et al 2007. It would be appropriate to also include a reference to previous work by O'Connell and White on C. elegans on Sas- 6, which was the first to show such maternal and paternal contribution forcentriolar components

Response: we have now included a reference to this earlier study.

© 2022. Published by The Company of Biologists under the terms of the Creative Commons Attribution License (https://creativecommons.org/licenses/by/4.0/).

Fourth decision letter

MS ID#: DEVELOP/2020/198762

MS TITLE: The centriolar satellite protein Cfap53/Ccdc11 facilitates the formation of the zygotic microtubule organizing center in the zebrafish embryo

AUTHORS: Sven Willekers, Federico Tessadori, Babet van der Vaart, Heiko H.W. Henning, Riccardo Stucchi, Maarten Altelaar, Bernard A.J. Roelen, Anna Akhmanova, and Jeroen Bakkers
ARTICLE TYPE: Research Article

I am happy to tell you that your manuscript has been accepted for publication in Development, pending our standard ethics checks.

© 2022. Published by The Company of Biologists under the terms of the Creative Commons Attribution License (https://creativecommons.org/licenses/by/4.0/).
